# Protective function of sclerosing cholangitis on IBD

**DOI:** 10.1136/gutjnl-2023-330856

**Published:** 2024-06-05

**Authors:** Tanja Bedke, Friederike Stumme, Miriam Tomczak, Babett Steglich, Rongrong Jia, Simon Bohmann, Agnes Wittek, Jan Kempski, Emilia Göke, Marius Böttcher, Dominik Reher, Anissa Franke, Maximilian Lennartz, Till Clauditz, Guido Sauter, Thorben Fründt, Sören Weidemann, Gisa Tiegs, Christoph Schramm, Nicola Gagliani, Penelope Pelczar, Samuel Huber

**Affiliations:** 1 I. Department of Medicine, Section of Molecular Immunology and Gastroenterology, University Medical Center Hamburg-Eppendorf, Hamburg, Germany; 2 Hamburg Center for Translational Immunology (HCTI), University Medical Center Hamburg-Eppendorf, Hamburg, Germany; 3 Center of Diagnostics, Institute of Pathology, University Medical Center Hamburg-Eppendorf, Hamburg, Germany; 4 Institute of Pathology with the Section Molecular Pathology and Cytopathology, University Medical Center Hamburg-Eppendorf, Hamburg, Germany; 5 I.Department of Medicine, University Medical Center Hamburg-Eppendorf, Hamburg, Germany; 6 Center for Experimental Medicine, Institute of Experimental Immunology and Hepatology, University Medical Center Hamburg-Eppendorf, Hamburg, Germany; 7 Martin Zeitz Center for Rare Diseases, University Medical Center Hamburg-Eppendorf, Hamburg, Germany

**Keywords:** INFLAMMATORY BOWEL DISEASE, Cholangitis, PRIMARY SCLEROSING CHOLANGITIS, COLONIC MICROFLORA

## Abstract

**Objective:**

There is a strong clinical association between IBD and primary sclerosing cholangitis (PSC), a chronic disease of the liver characterised by biliary inflammation that leads to strictures and fibrosis. Approximately 60%–80% of people with PSC will also develop IBD (PSC-IBD). One hypothesis explaining this association would be that PSC drives IBD. Therefore, our aim was to test this hypothesis and to decipher the underlying mechanism.

**Design:**

Colitis severity was analysed in experimental mouse models of colitis and sclerosing cholangitis, and people with IBD and PSC-IBD. Foxp3^+^ Treg-cell infiltration was assessed by qPCR and flow cytometry. Microbiota profiling was carried out from faecal samples of people with IBD, PSC-IBD and mouse models recapitulating these diseases. Faecal microbiota samples collected from people with IBD and PSC-IBD were transplanted into germ-free mice followed by colitis induction.

**Results:**

We show that sclerosing cholangitis attenuated IBD in mouse models. Mechanistically, sclerosing cholangitis causes an altered intestinal microbiota composition, which promotes Foxp3^+^ Treg-cell expansion, and thereby protects against IBD. Accordingly, sclerosing cholangitis promotes IBD in the absence of Foxp3^+^ Treg cells. Furthermore, people with PSC-IBD have an increased Foxp3^+^ expression in the colon and an overall milder IBD severity. Finally, by transplanting faecal microbiota into gnotobiotic mice, we showed that the intestinal microbiota of people with PSC protects against colitis.

**Conclusion:**

This study shows that PSC attenuates IBD and provides a comprehensive insight into the mechanisms involved in this effect.

**WHAT IS ALREADY KNOWN ON THIS TOPIC**

- There is a strong clinical association between IBD and primary sclerosing cholangitis (PSC). However, currently it is unknown, if this association is due to common genetic polymorphisms or if PSC may drive IBD.

**WHAT THIS STUDY ADDS**

- Unexpectedly, we found that PSC attenuates IBD. Mechanistically, PSC causes an altered intestinal microbiota composition, which promotes Foxp3^+^ Treg-cell expansion, and thereby protects against IBD.

**HOW THIS STUDY MIGHT AFFECT RESEARCH, PRACTICE OR POLICY**

- We believe that our data build a basis for the development of new therapeutical strategies targeting the microbiota-Foxp3^+^ Treg-cell axis in IBD.

## Introduction

IBD is characterised by chronic relapsing intestinal inflammation. The exact aetiology of IBD is not completely understood, but it is known that IBD is characterised by chronic inflammation, intestinal dysbiosis and mucosal barrier defects. Thus, one hypothesis is that IBD is a result of an aberrant immune response against intestinal bacteria in genetically susceptible individuals.^1–3^ There is a strong clinical association between IBD and primary sclerosing cholangitis (PSC), a chronic, cholestatic liver disease characterised by inflammation and fibrosis of the bile ducts inside and outside the liver. Approximately 60%–80% of people with PSC have concomitant IBD (from here on referred to as PSC-IBD).^1 4^ Conversely, only about 5% of people with IBD will develop PSC during their disease course.^2^ Notably, people suffering from PSC-IBD have a phenotype distinct from Crohn’s disease (CD) and Ulcerative colitis (UC), characterised by an overall milder IBD severity, a higher prevalence of right-sided predominant pancolitis, rectal sparing, backwash ileitis and an increased risk of developing colorectal neoplasia.^3 5^ Even for people with PSC without clinically manifested IBD, we have previously shown that a high proportion exhibits molecular signs of intestinal inflammation, characterised by immune cell infiltration and expression of proinflammatory cytokines in intestinal biopsies.^6^

The factors that contribute to the development of PSC-IBD are not yet understood. Previous studies suggest a critical role of CD4^+^ Foxp3^+^ regulatory T cells (Foxp3^+^ Treg) in IBD, as well as PSC.^7–9^ In line with these data, reduced Foxp3^+^ Treg-cell numbers and function were associated with single nucleotide polymorphisms in the *IL2RA* gene present in people with IBD and PSC.^10–12^ Interestingly, the microbiota plays a key role in the emergence of Foxp3^+^ Treg cells: microbiota-derived short-chain fatty acids (SCFAs) can facilitate the induction of Foxp3^+^ Treg cells both in *in vitro* and in animal models.^13 14^ Accordingly, aside from genetic predispositions in genes regulating Foxp3^+^ Treg-cell function, the intestinal microbiota has been suggested to be one of the contributing factors for the close association of IBD and PSC.^15^ Indeed, both IBD and PSC are characterised by intestinal dysbiosis. Moreover, direct comparisons revealed distinct microbiota compositions between these diseases.^16–22^ Thus, among others, the phylae *Veillonella* and *Escherichia* have been reported to be enriched in people with PSC-IBD compared with IBD alone, that are proposed to promote immune cell migration to the gut. In addition, bacteria of the *Lachnospiraceae* family which produce anti-inflammatory SCFAs were reported to be increased in people with PSC.^23^ However, it remains unclear, whether changes in the microbial composition caused by PSC lead to an altered Foxp3^+^ Treg-cell expansion and function that contributes to the phenotype of IBD in people with PSC.

Taken together, there is a clear connection between IBD and PSC. However, whether PSC increases the risk for IBD but attenuates its phenotype remains to be elucidated. In this study we combined cellular and microbial analyses from experimental mouse models of colitis and sclerosing cholangitis, biopsies and stool samples of people with PSC-IBD and IBD, and then performed human faecal microbiota transplantation (FMT) into gnotobiotic mice to decipher the impact of PSC on IBD.

## Results

### Experimental sclerosing cholangitis attenuates colitis severity and increases Foxp3^+^ Treg-cell frequency in mice

**Figure 1:**
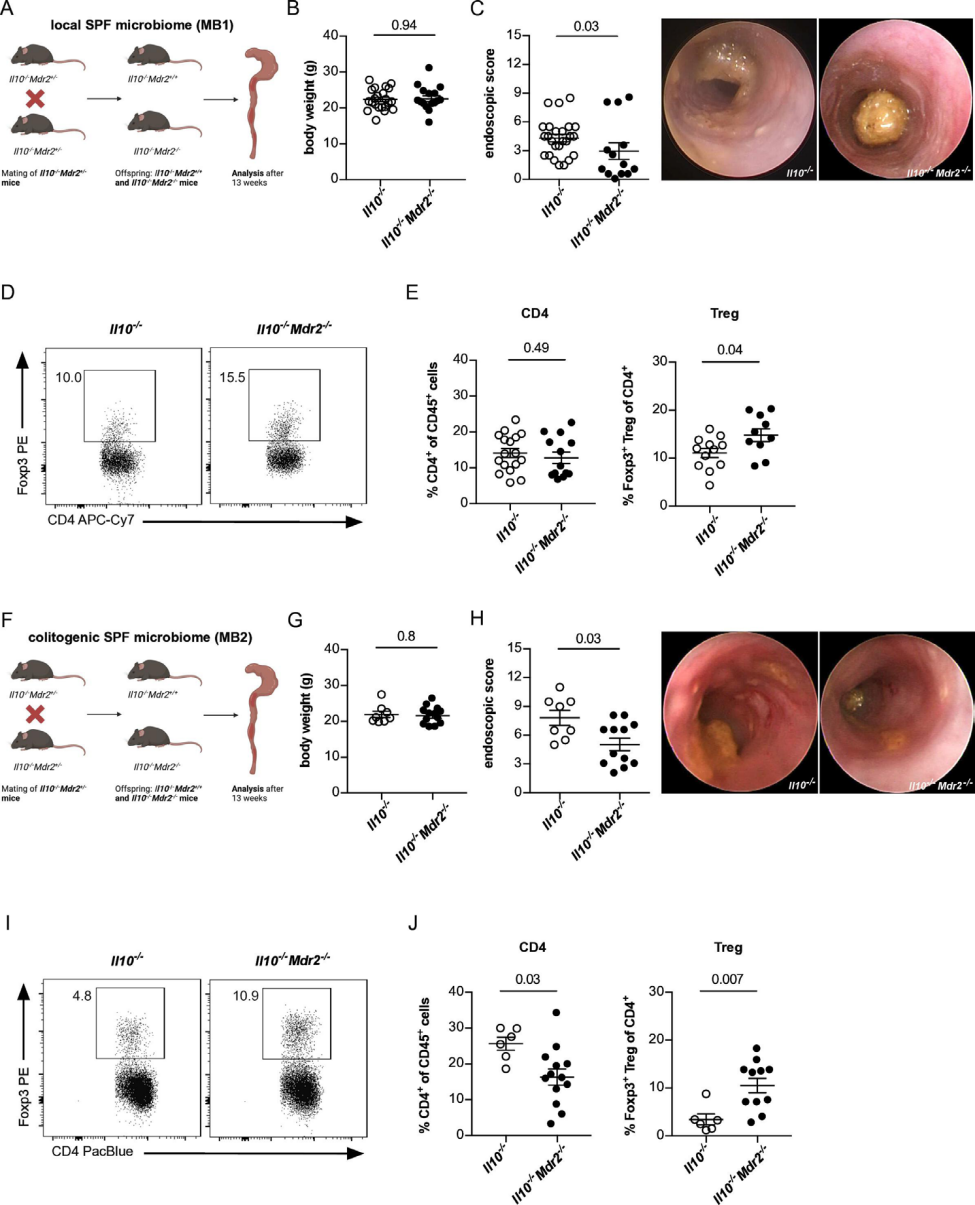
Spontaneous colitis is reduced in mice with concomitant experimental primary sclerosing cholangitis in *Il10^*−/−*^Mdr2^*−/−*^* mice. (A) Graphical breeding scheme for generation of *Il10^*−/−*^* and *Il10^*−/−*^Mdr2^*−/−*^* littermates. Mice were bred under specific pathogen-free (SPF) conditions in the local mouse facility (MB1). After weening, litters were separated with respect to their genotype. At an age of 12 weeks, (B) body weight (n=22 *Il10^*−/−*^*, n=16 *Il10^*−/−*^Mdr2^*−/−*^*) and (C) colon inflammation was assessed by mouse colonoscopy (n=25 *Il10^−/−^*, n=13 *Il10^−/−^Mdr2^−/−^*), as described in material and methods. (D, E) Flow cytometry analysis of colon infiltrating CD4^+^ T-cell (n=17 *Il10^−/−^*, n=13 *Il10^−/−^Mdr2^−/−^*) and Foxp3^+^ Treg-cell frequencies of 12 weeks old mice (n=12 *Il10^−/−^*, n=10 *Il10^−/−^Mdr2^−/−^*). (F) Graphical breeding scheme for generation of *Il10^−/−^* and *Il10^−/−^Mdr2^−/−^* littermates bred in the presence of a colitogenic SPF microbiome (MB2) containing *Helicobacter hepaticus*, that was transferred to the founding animals. After weening, litters were separated with respect to their genotype. At the age of 12 weeks (G) body weight (n=8 *Il10^−/−^*, n=13 *Il10^−/−^Mdr2^−/−^*), (H) colonoscopy (n=8 *Il10^−/−^*, n=12 *Il10^−/−^Mdr2^−/−^*) and (I, J) frequencies of colon infiltrating CD4^+^ T cells and Foxp3^+^ Treg cells (n=6 *Il10^−/−^*, n=11 *Il10^−/−^Mdr2^−/−^*) were analysed. For statistical analysis, Mann-Whitney U test was performed.

First, we aimed to test the connection between IBD and sclerosing cholangitis in experimental mouse models. To this end, *Il10^−/−^* mice, which develop spontaneous colitis^24^ were crossed to *Mdr2^*−/−*^* mice, a mouse model for experimental sclerosing cholangitis^25^ (figure 1A). As expected, *Il10^*−/−*^Mdr2^*−/−*^* mice, but not *Il10^*−/−*^* mice, developed sclerosing cholangitis based on increased transaminase AST and ALT levels, and fibrosis score (online supplemental figure S1A,B). Next, we assessed IBD severity. We found that *Il10^*−/−*^* and *Il10^*−/−*^Mdr2^*−/−*^* mice developed an overall mild colitis (figure 1B,C). Interestingly, *Il10^*−/−*^Mdr2^*−/−*^* mice with a concomitant experimental sclerosing cholangitis developed significantly reduced colitis compared with *Il10^*−/−*^* mice (figure 1C). However, there was little impact on weight, despite the differences observed in colitis severity using endoscopy. Of note, we aimed to induce a mild to moderate colitis severity in our experiments in order to limit the suffering of the animals. Thus, all mice showed a relatively mild weight loss, and we therefore may have not observed a difference. Moreover, while colonic CD4^+^ T-cell infiltration was comparable between the groups (figure 1D), the proportion of Foxp3^+^ Treg cells within the CD4^+^ T-cell population was significantly increased in the inflamed colon of *Il10^*−/−*^Mdr2^*−/−*^* compared with *Il10^*−/−*^* mice (figure 1D,E).

10.1136/gutjnl-2023-330856.supp2 Supplementary data

The intestinal microbiota composition is known to impact colitis-susceptibility^26^. Therefore the colitis development we observed in *Il10^−/−^* mice under specific pathogen-free (SPF) conditions of the local mouse facility (referred to as MB1) was generally mild. To this end, we next aimed to determine spontaneous colitis development in *Il10^−/−^* mice bred in the presence of a colitogenic SPF microbiota, that showed a distinct beta diversity compared with MB1, including an enrichment of *Helicobacter* on genus level (referred to as MB2) (online supplemental figure S2A,B; figure 1F). As expected, the mice bred under MB2 conditions showed an overall increased susceptibility to developing colitis compared with mice with MB1 microbiota (figure 1C,H). Comparisons between *Il10^−/−^* and *Il10^−/−^Mdr2^−/−^* mice bred under MB2 conditions revealed no differences in body weight between the groups (figure 1G). However, colitis severity in *Il10^−/−^Mdr2^−/−^* mice with concomitant sclerosing cholangitis (online supplemental figure S1C,D) was significantly reduced compared with *Il10^−/−^* mice (figure 1H). Moreover, *Il10^−/−^Mdr2^−/−^* mice bred under MB2 condition showed reduced colonic CD4^+^ T-cell infiltration and increased Foxp3^+^ Treg-cell accumulation compared with *Il10^−/−^* mice (figure 1I,J).

**Figure 2:**
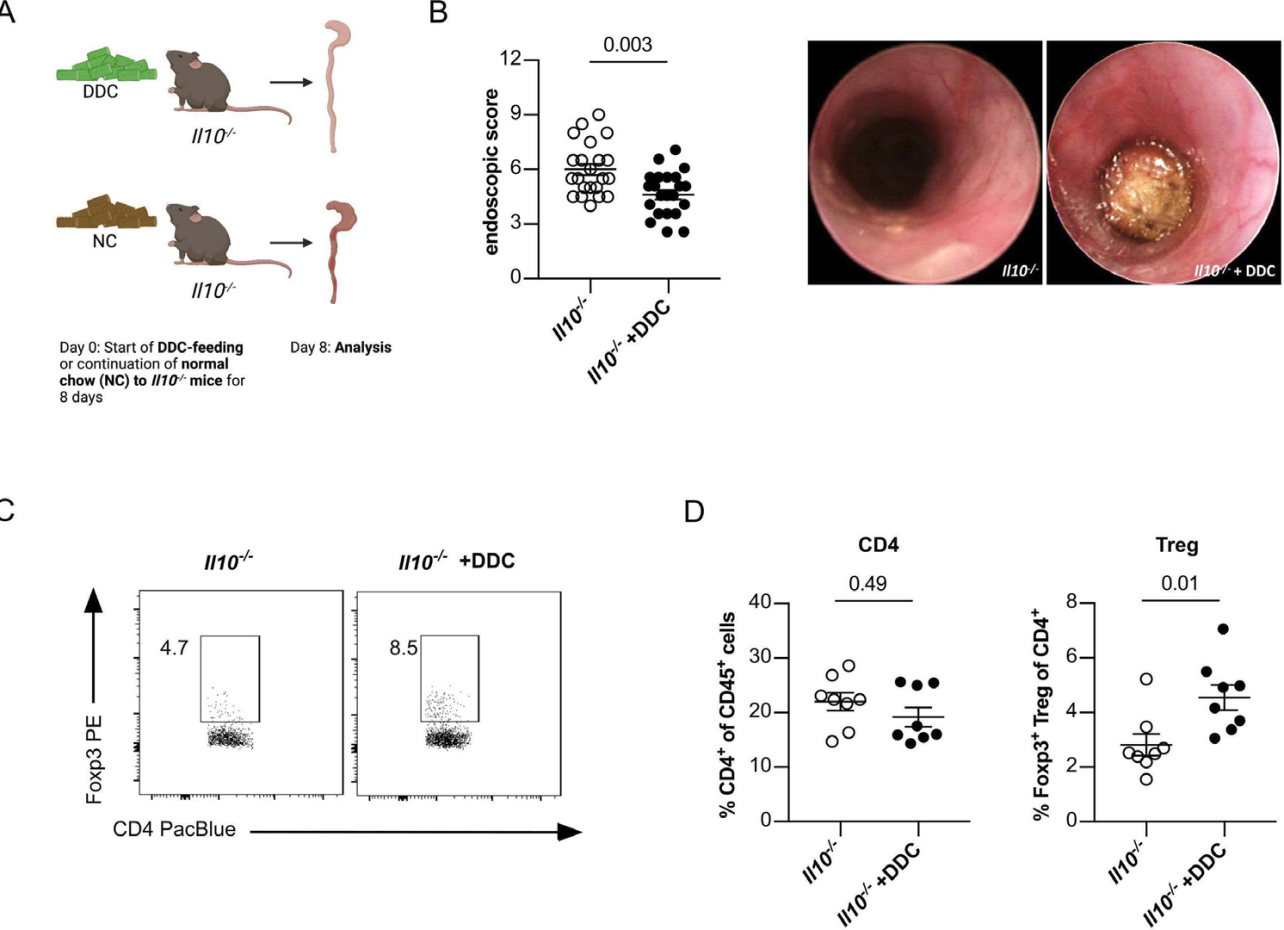
Spontaneous colitis is reduced in *Il10^−/−^* mice with concomitant 3,5-diethoxycarbonyl-1,4-dihydrocollidine (DDC)-mediated liver cholestasis. (A) Graphical scheme of the experimental setup. At an age of 6–8 weeks *Il10^−/−^* mice were gavaged with MB2. Four weeks after reconstitution, liver cholestasis was induced by 0.1% DDC feeding supplemented into the normal chow diet. After 8 days, (B) colonic inflammation was analysed by mouse colonoscopy (n=22 mice per group). (C, D) On day 9, mice were sacrificed and frequencies of colon infiltrating CD4^+^ T cells and Foxp3^+^ Treg cells were analysed using flow cytometry (8=mice per group). For statistical analysis Mann-Whitney U test was performed.

Next, we aimed to validate our observation in *Il10^−/−^Mdr2^−/−^* mice using a second model of experimental sclerosing cholangitis. To this end, we fed *Il10^−/−^* mice a 3,5-diethoxycarbonyl-1,4-dihydrocollidine (DDC) diet.^27^ We used mice with the more colitogenic MB2 microbiota (figure 2A). DDC diet-induced sclerosing cholangitis in *Il10^−/−^* mice as determined by blood transaminase levels and fibrosis development (online supplemental figure S3A,B). In line with our results in *Il10^−/−^Mdr2^−/−^* mice, colitis severity and CD4^+^ T-cell infiltration in the inflamed colon of *Il10^−/−^* mice was attenuated under the DDC diet (figure 2B), and frequencies of colonic Foxp3^+^ Treg cells were increased compared with the regular chow diet (figure 2C,D).

Thus, sclerosing cholangitis attenuates colitis severity in mouse models and is associated with an increased colonic Foxp3^+^ Treg-cell frequency.

### Attenuated colitis severity in mice with sclerosing cholangitis is dependent on Foxp3^+^ Treg cells

**Figure 3:**
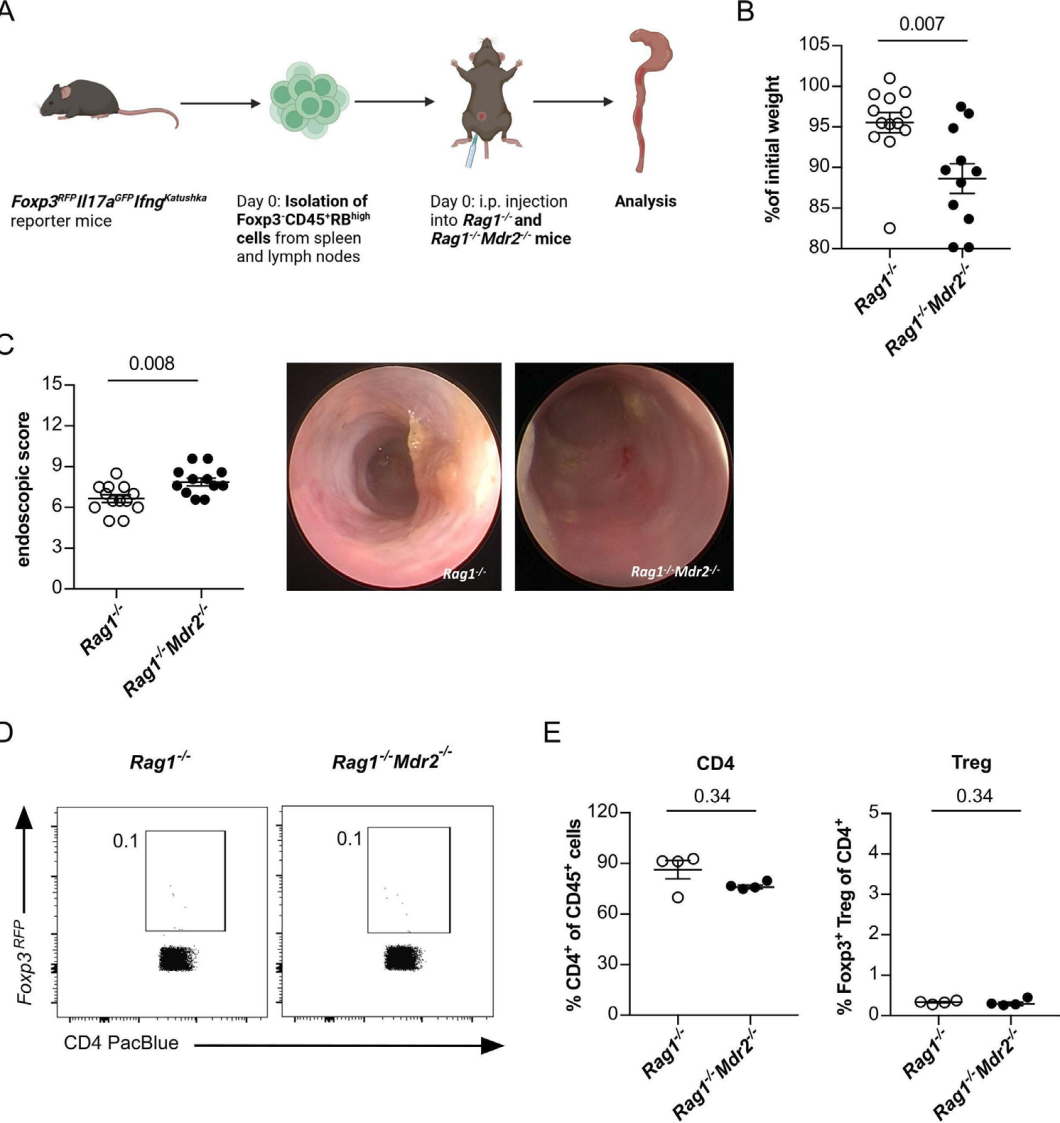
Increased colitis manifestation in *Rag1^−/−^Mdr2^−/−^* mice after Foxp3^−^CD45RB^high^ T-cell transfer. (A) Graphical scheme of the experimental setup. (B) At an age of 8–10 weeks *Rag1^−/−^* and *Rag1^−/−^Mdr2^−/−^* mice were gavaged with MB2. After 4 weeks of reconstitution, colitis was induced on transfer of flow cytometry sorted Foxp3^−^CD45RB^high^ CD4^+^ T cells, isolated from *Foxp3-RFP* reporter mice. After 13 days of T-cell reconstitution, (B) weight loss and (C) colonic inflammation by colonoscopy were analysed (n=13 *Rag1^−/−^*, n=12 *Rag1^−/−^Mdr2^−/−^*). (D, E) At day 14, mice were sacrificed and frequencies of colon infiltrating CD4^+^ T cells and Foxp3^+^ Treg cells were analysed by flow cytometry in one of three experiments (n=4 *Rag1^−/−^* n=4 *Rag1^−/−^Mdr2^−/−^*). For statistical analysis Mann-Whitney U test was performed.

Since the reduced colitis severity was associated with a shift of CD4^+^ T-cell infiltration towards Foxp3^+^ Treg cells, we hypothesised that Foxp3^+^ Treg cells contribute to the limitation of colonic inflammation. Foxp3^+^ Treg cells are well known for their capacity to limit intestinal inflammation and restore immune homeostasis.^28^ Thus, to define the contribution of Foxp3^+^ Treg cells to the PSC-mediated attenuation of colitis, we used the T-cell transfer colitis model, in which Foxp3^+^ Treg cells are largely absent.^29^ To that end, we induced colitis in lymphopenic *Rag1^−/−^* and *Rag1^−/−^Mdr2^−/−^* mice, by transfer of naïve CD4^+^Foxp3^−^CD45RB^high^ cells (figure 3A). As expected, *Rag1^−/−^Mdr2^−/−^* mice, but not *Rag1^−/−^* mice developed concomitant sclerosing cholangitis (online supplemental figure S4,B). Next, we assessed colitis severity and found it not to be attenuated in *Rag1^−/−^Mdr2^−/−^* mice, but in fact to be significantly increased compared with *Rag1^−/−^* based on weight loss and endoscopic score (figure 3B,C). As expected, no considerable Foxp3^+^ Treg-cell levels were detectable among CD4^+^ T-cell infiltrating cells (figure 3D,E).

Taken together, the protective effect of sclerosing cholangitis on colitis appears to be dependent on the presence of Foxp3^+^ Treg cells.

### FMT from *Mdr2^−/−^* mice into germ-free wild-type mice attenuates colitis severity

Alterations in the intestinal microbiota are a hallmark of IBD.^30^ Moreover, the intestinal microbiota is known to impact Foxp3^+^ Treg-cell differentiation and expansion.^31^ We therefore hypothesised that sclerosing cholangitis may alter the intestinal microbiota, and thus, reduce colitis severity. In order to test this hypothesis, we profiled the microbiota of stool samples collected from mice suffering from colitis alone (eg, *Il10^−/−^* mice and *Rag1^−/−^* mice on colitis induction via transfer of CD45Rb^high^ cells) and with concomitant sclerosing cholangitis (eg, *Il10^−/−^Mdr2^−/−^* mice and *Rag1^−/−^Mdr2^−/−^* mice on colitis induction via transfer of CD45Rb^high^ cells) (online supplemental figure S5). Comparison of beta diversities revealed clustering with some overlap of both experimental groups (online supplemental figure S5A,C), although a spread of samples between the groups was detected in both models. Of note, on the genus level, we found several taxa that significantly differed in abundance between the groups in the transfer colitis model (online supplemental figure S5D), but only one taxon in the *Il10^−/−^* model (online supplemental figure S5B). Most notably, an enrichment of genera of the *Lachnospiraceae* family was found in stool samples of mice suffering from colitis with concomitant liver inflammation in transfer colitis (online supplemental figure S5D).

**Figure 4:**
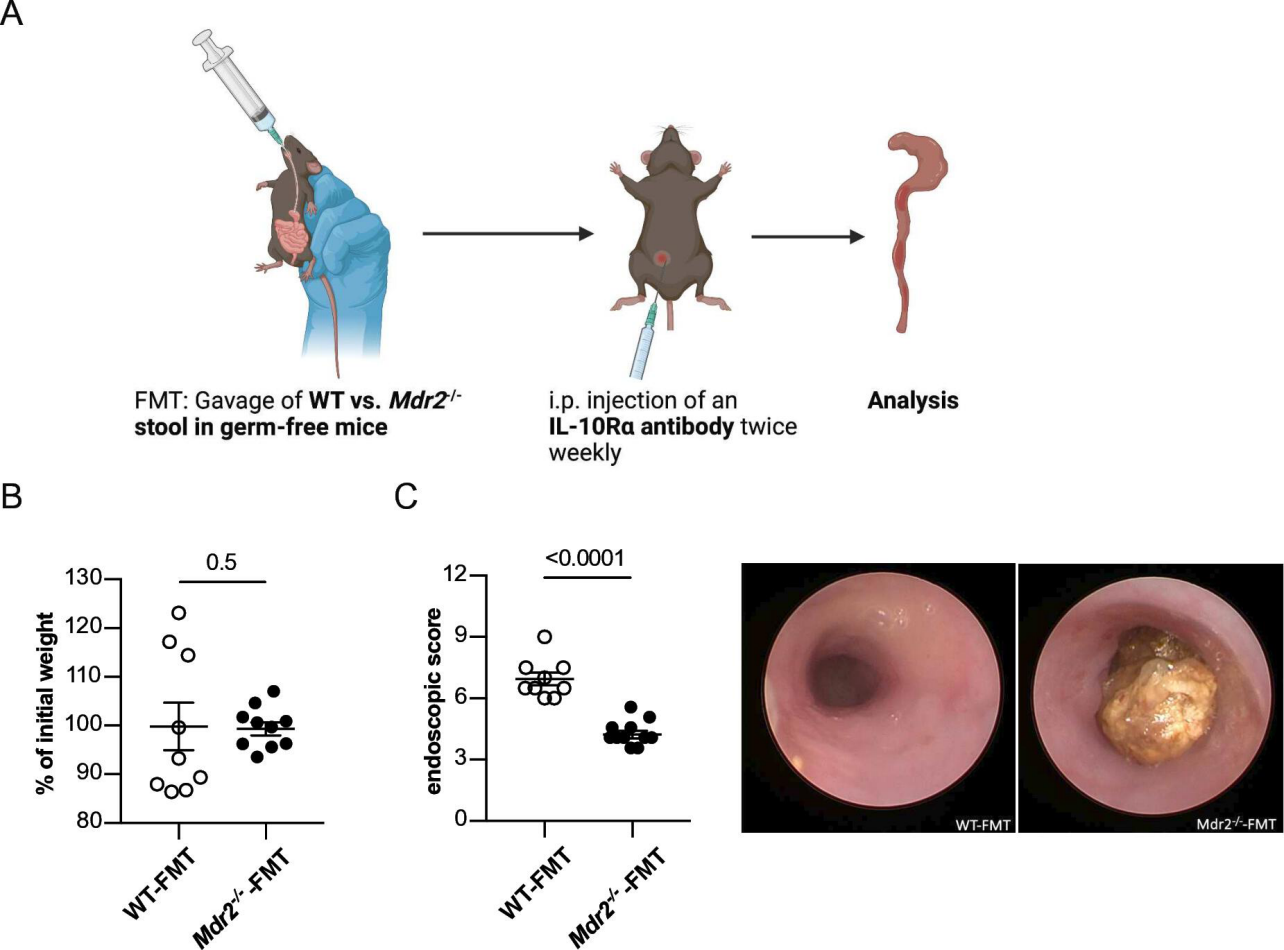
Reduced colitis severity in germ-free wild-type mice after transfer of *Mdr2^−/−^* microbiota. (A) Graphical scheme of the experimental procedure. In brief, faecal microbiota obtained from wild-type and *Mdr2^−/−^* mice, harbouring MB2 microbiome, was gavaged into germ-free wild-type mice. One day later, colitis was induced in these mice by intraperitoneal injection of 0.25 mg anti-IL10Rα antibody per mouse two times a week. After 13 days of colitis induction, (B) weight loss was determined and (C) colonic inflammation was analysed by colonoscopy (n=9 WT-FMT, n=10 *Mdr2^−/−^*-FMT). FMT, faecal microbiota transplantation; WT, wild-type.

To decipher the functional relevance of the observed PSC-induced microbiota alterations on colitis severity, we next reconstituted germ-free wild-type mice with stool derived from mice with sclerosing cholangitis (*Mdr2^−/−^* mice) or without sclerosing cholangitis (wild-type mice), respectively, and induced colitis in these mice using a blocking anti-IL10Rα mAb^32^ (figure 4A). In accordance with our above-mentioned results (figure 1B,G), a mild weight loss was observed on colitis induction, that did not differ between the groups (figure 4B). However, endoscopic colitis severity was reduced in germ-free mice reconstituted with microbiota derived from *Mdr2^−/−^* mice compared with wild-type mice (figure 4C).

Taken together, these results indicate that sclerosing cholangitis leads to alterations in the intestinal microbiota, in particular to an enrichment in genera of the *Lachnospiraceae* family. Furthermore, this altered intestinal microbiota of *Mdr2^−/−^* mice suffering from sclerosing cholangitis is protective against colitis, when compared with wild-type mice.

### Colitis severity in germ-free mice is attenuated after FMT from people with PSC-IBD compared with IBD

**Figure 5:**
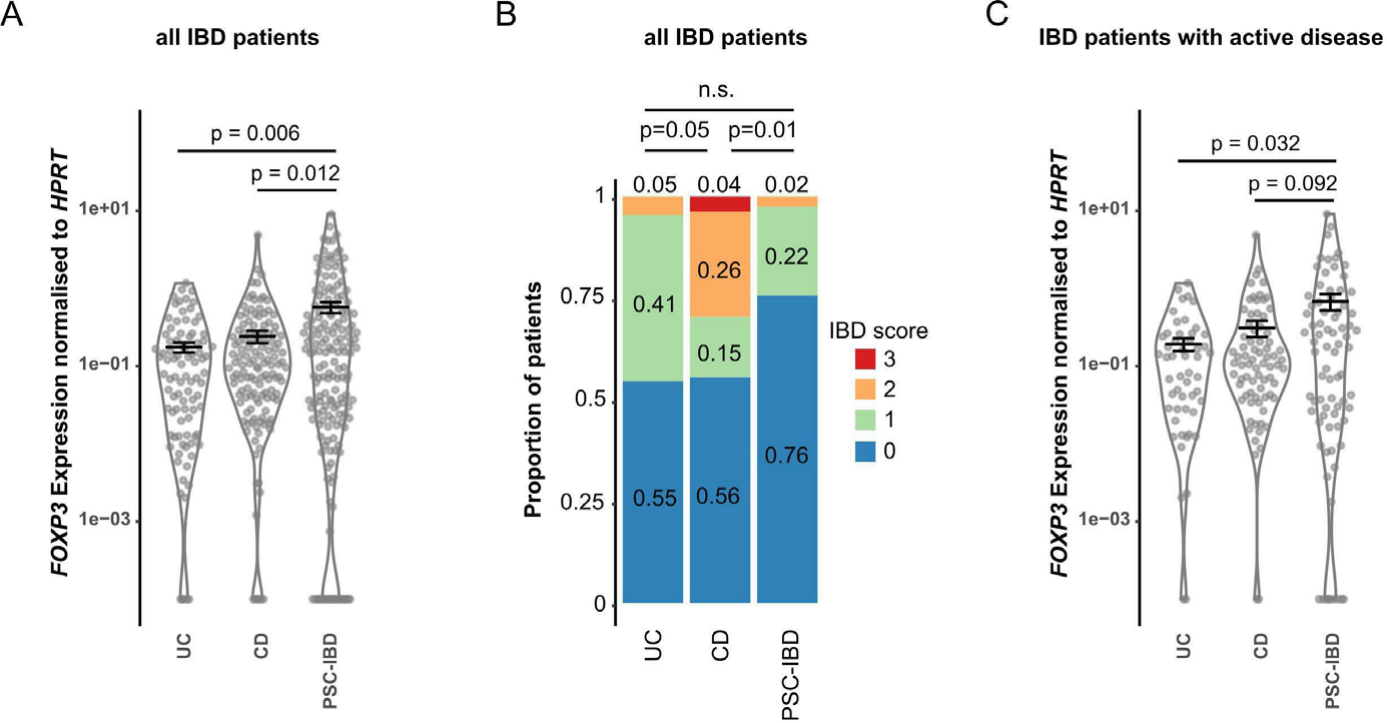
*FOXP3* mRNA expression and endoscopic IBD scoring reveal reduced clinical manifestation of IBD in people with primary sclerosing cholangitis (PSC-IBD). Description of a cohort including 29 people with Crohn’s disease (CD), 22 with Ulcerative colitis (UC) and 41 with PSC-IBD. (A) *FOXP3* mRNA expression levels were analysed from intestinal biopsies taken from the terminal ileum, ascending and descending colon and sigma/rectum from every person. (B) IBD severity was determined based on CDAI (persons with CD) and Mayo score (all other persons). Both scores were merged into a unified IBD score (healthy/remission: 0, mild: 1, moderate: 2, severe: 3 points). (C) *FOXP3* mRNA expression levels were analysed from intestinal biopsies taken from the terminal ileum, ascending and descending colon and sigma/rectum from every person with clinically active disease. To test for significance MLEM, post hoc Dunnett test was used for (A and C). Fisher’s exact test was used for (B).

Based on the data obtained in the murine system, we next characterised *FOXP3* mRNA expression levels in intestinal biopsies taken from a cohort of people with CD (n=29), UC (n=22) and PSC-IBD (n=41). We observed increased *FOXP3* mRNA expression in the intestinal tissue of people with PSC-IBD compared with both, individuals with CD and UC (figure 5A). Within the cohort, we found milder IBD severity in people with concomitant PSC compared with people with CD and to a lesser extent to people with UC, as described previously (figure 5B).^5^ To account for this bias in disease severity, we next compared only those individuals with a clinically active disease as assessed by their physician. We again found an increased *FOXP3* mRNA expression in the intestinal tissue of people with PSC-IBD compared with both individuals with CD and UC (figure 5C). Of note, the mean IBD score in all three groups was low and comparable (mean IBD-score for PSC-IBD: 0.48, CD: 0.52, UC: 0.78). To further test, if this decrease is biased by biopsies from a certain location, we plotted all biopsies from the same location for all patients. We found the same trend in all locations analysed: individuals with PSC-IBD having a higher *FOXP3* mRNA expression compared with people with IBD without PSC (online supplemental figure S6A, online supplemental table S2). Next, we measured FOXP3 protein levels in tissue sections using immunohistochemistry. To this end we focused on biopsies from the terminal ileum and sigma/rectum. In line with the mRNA expression, we found an increased number of FOXP3^+^ cells in people with PSC-IBD compared with IBD without PSC (online supplemental figure S6B,C).

10.1136/gutjnl-2023-330856.supp4 Supplementary data

**Figure 6:**
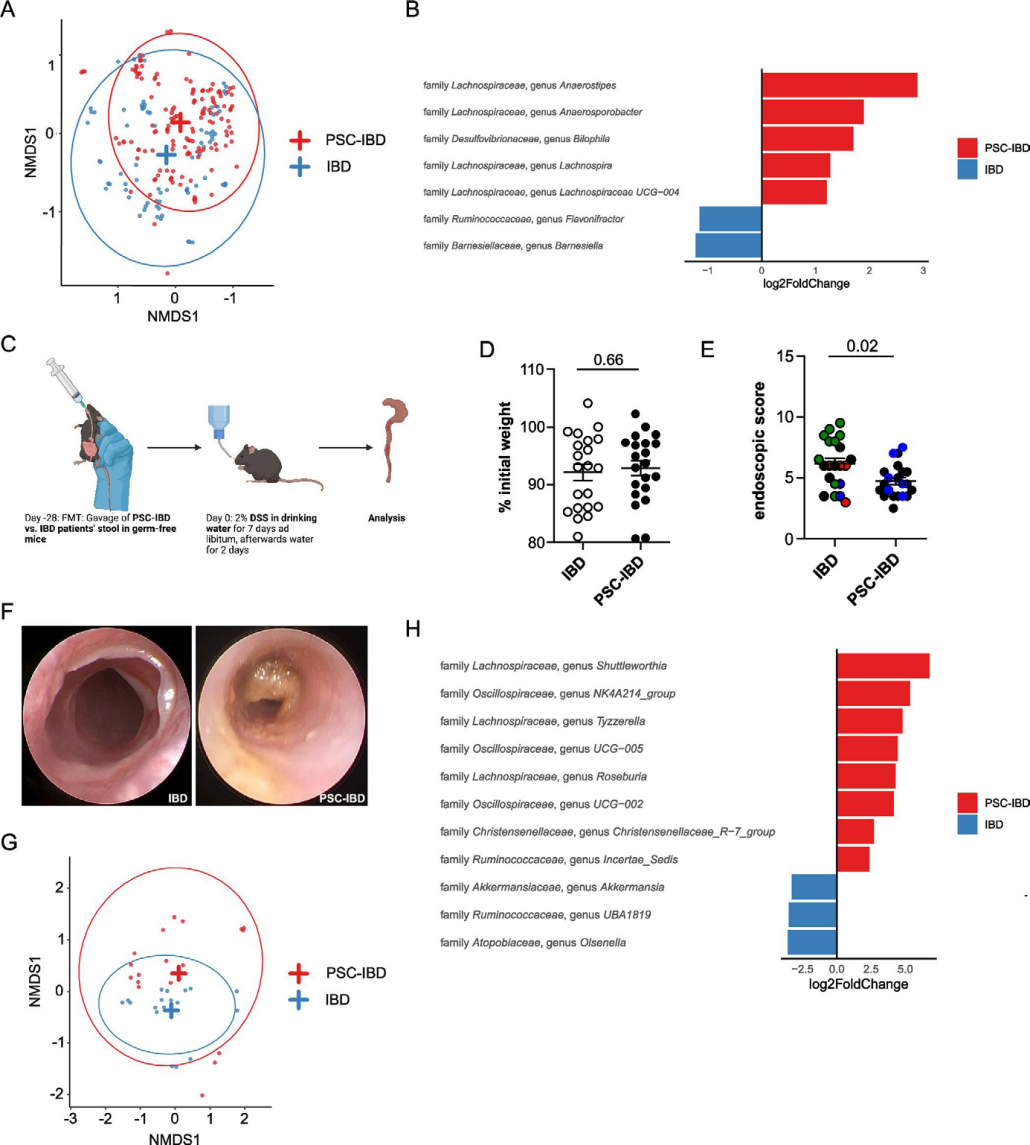
Colitis severity in germ-free mice is attenuated after FMT from people with primary sclerosing cholangitis and colitis (PSC-IBD), enriched for genera of the *Lachnospiraceae* family. Microbiota profiling was performed on mucosal tissue samples of our IBD and PSC-IBD cohort, as described in the material and methods. (A) PCoA of Bray-Curtis dissimilarities shows beta diversity across people with IBD and PSC-IBD. (B) Genera with significantly different abundance between people with IBD and PSC-IBD. (C) Graphical scheme of the protocol for faecal microbiota transplantation of stool derived from IBD or PSC-IBD patients into germ-free wild-type mice, and subsequent DSS colitis induction. After 9 days of colitis induction, (D) weight loss was determined and (E and F) colonic inflammation was analysed by colonoscopy (each dot represents one mouse). IBD activity of the donor is shown as: remission (black), mild (green), moderate (blue) and severe (red). (G and H) Microbiota profiling from stool samples collected from mice after reconstitution with stool samples from our IBD and PSC-IBD cohort. (D–H) n=21 mice transplanted with IBD stool; n=21 mice transplanted with PSC-IBD stool were used in four independent experiments.

Next, we aimed to test whether the microbiota from people with PSC would protect against the development of concomitant IBD. Thus, we first performed microbiota profiling of mucosa-adherent bacteria isolated from intestinal biopsies derived from our IBD and PSC-IBD cohort, that has been partially published in Wittek et al, 2023. Sequencing of faecal microbiota revealed a large overlap, but also some differences in the microbiota composition of people with IBD and those with PSC-associated IBD. However, it is important to note that our study was not powered to decipher detailed microbiota differences between PSC-IBD and IBD as this point has been addressed by previous larger studies.^23 33^ Beta diversity comparison revealed a large overlap between people with IBD and PSC-IBD (figure 6A). In fact, on the genus level only a few taxa differed in abundance between both groups (figure 6B). Interestingly, genera of the *Lachnospiraceae* family were enriched in intestinal biopsies from people with PSC-IBD compared with IBD. To test the functional relevancy of this finding, we reconstituted germ-free wild-type mice with faecal microbiota samples derived from people with IBD or PSC-IBD and induced DSS colitis on reconstitution (figure 6C). Weight loss was comparable in both groups (figure 6D). However, the colitis severity as assessed by endoscopy was significantly reduced in mice reconstituted with faecal microbiota from people with PSC-IBD compared with IBD alone (figure 6E,F). To address whether ursodeoxycholic acid (UDCA) treatment mediates the observed effect, we performed a gnotobiotic mouse experiment. Specifically, a faecal microbiota transfer from healthy control (HC), without UDCA treatment, and primary biliary cholangitis (PBC) patients, with UDCA treatment, into germ-free mice was performed. People with PBC with mild cholestasis comparable to that of people with PSC-IBD were selected as cholestatic controls (online supplemental figure S7A,B). On engraftment, DSS-colitis was induced. A comparable colitis severity was observed between these groups, indicating that UDCA does not *per se* influence the colitis activity (online supplemental figure S7C,D). Next, we analysed whether the observed protection of these PSC-IBD-specific gnotobiotic mice is associated with an enrichment of genera of the *Lachnospiraceae* family on FMT. Microbiota profiling of donors (see online supplemental table S1 for the clinical information) and recipient mice was performed and can be found in online supplemental figure S8A. A Permanova analysis showed a significant contribution of disease, group (donor vs recipient) and donor on the variation observed in the data (online supplemental figure S8B). The abundance of bacteria in the different donors is comparable to a cross-sectional cohort (online supplemental figure S8C). When looking at the 10 highest abundant families, we observed that these were in most cases distributed similarly between recipients of the same donor, although at different proportions compared with the donor (online supplemental figure S8D). Beta diversities showed some overlapping of clusters representing each of the groups (figure 6G). Indeed, a strong enrichment of genera of the *Lachnospiraceae* family was detectable in faecal samples of mice that had been reconstituted with PSC-IBD stool compared with IBD stool (figure 6H).

10.1136/gutjnl-2023-330856.supp3 Supplementary data

In conclusion, these data indicate that PSC induces alterations of the intestinal microbiota, in particular an enrichment of genera of the *Lachnospiraceae* family, which in turn attenuate colitis susceptibility.

## Discussion

In line with previous reports,^3 5^ we found that people with PSC-IBD present with milder colitis severity compared with people with IBD without PSC in our cohort. Likewise, we found a lower IBD susceptibility in a genetic (*Mdr2^−/−^*) and an induced (DDC-diet) mouse model of sclerosing cholangitis.

Alterations in the intestinal microbiota of people with PSC-IBD and IBD without PSC have been documented in various studies. These studies have yielded somewhat divergent findings,^19 33^ possibly due to variations in participant selection criteria, sampling locations and sample processing. Our study, along with several others, consistently identified an elevation in *Lachnospiraceae* among people with PSC-IBD compared with those with IBD without PSC.^17 23^ It could be possible that cholestasis, which is observed in people with PSC mediates the observed effects on the intestinal microbiota. In this case a similar effect should be observed in people and mouse models with cholestasis even in the absence of PSC. Further studies will be critical to address this point.

We observed that the protective effect of sclerosing cholangitis on colitis susceptibility was transferable on faecal microbiota transfer from *Mdr2^−/−^* mice and people with PSC-IBD into germ-free mice. Overall, we found genera of the *Lachnospiraceae* family to be abundant in the faecal samples of people with PSC used for the faecal microbiota transfer experiment. This finding is in line with a previous study by our group.^6^ Importantly, genera of the *Lachnospiraceae* family were over-presented in faecal samples after engraftment of the germ-free mice, supporting the notion that these could be involved in the protective effect. However, there is still the limitation that the number of donors may not fully capture the range of microbiota variability in people with PSC. In line with this finding, a previous publication has reported that *Mdr2^−/−^* mice treated with vancomycin, which reduced *Lachnospiraceae* and *Clostridiaceae*, had an increased liver pathology. Supplementation of these mice after antibiotic treatment with a 23 strain *Lachnospiraceae* consortium reduced histological liver inflammation and fibrosis.^22^ Conversely, in people with PSC, the *Lachnospiraceae Blautia* (genus), *Lachnospiraceae bacterium 1_4_56FAA* was negatively correlated with the Mayo risk score.^22^

Another important observation of this study is the association between increased Foxp3^+^ Treg-cell accumulation in the colon and over-representation of *Lachnospiraceae* in faecal samples. This has been observed in our mouse models of experimental sclerosing cholangitis with concomitant colitis, and in people with PSC-IBD compared with people suffering from IBD without PSC. *Lachnospiraceae* have indeed been associated with the production of SCFAs,^34 35^ which in turn have been linked to the induction of Foxp3^+^ Treg cells.^35–38^ Therefore, further assessment of SCFAs from faecal samples of our IBD and PSC-IBD cohort and mouse models of sclerosing cholangitis is required to test whether the enrichment in *Lachnospiraceae* is indeed associated with increased SCFA levels and subsequently increased Foxp3^+^ Treg-cell numbers.

One limitation of this study is that the role of *Lachnospiraceae* remains controversial. While some taxa produce butyrate, which can strengthen the intestinal barrier, others produce propionate, which can drive mucin degradation.^34^ More in-depth analysis of this family of bacteria in people with PSC-IBD, for example, through metagenomics, could help to identify which taxa are involved and how their metabolites could influence IBD development. Similarly, another publication^39^ showed an increase of *Lachnospiraceae* in faecal samples of *Mdr2^−/−^* mice. Transfer of the dysbiotic *Mdr2^−/−^* microbiota into healthy wild-type mice induced NLRP3 activation in the gut and the liver, which sustained liver injury and promoted disease progression. It would be important to further investigate the role of different taxa of *Lachnospiraceae* in the relationship of PSC and IBD.

Interestingly, a recent study identified *Klebsiella pneumoniae* in mesenteric lymph nodes of people with PSC, and also in faecal samples.^40^ Subsequent studies revealed that *K. pneumoniae* causes disruption in the epithelial barrier, resulting in the translocation of bacteria and subsequent inflammation in the liver. These discoveries emphasise how pathobionts contribute to dysfunction in the intestinal barrier and inflammation in the liver.^40^ Given the crucial role of the microbiome, it would be of interest to study whether the PSC microbiota can also modify complications of IBD in PSC, such as cancer risk.

The observation that people with PSC-IBD have a lower IBD activity on average, compared with people with IBD,^6^ is also reflected in our selected donors for the faecal microbiota experiments (online supplemental table S1). However, it appears that the observed protective effect was not linked to this difference. Of note, neither *Mdr2*-deficient mice nor control mice, which were used as donors for the FMT experiment, developed spontaneous colitis. This strengthens the observation that the IBD activity of the microbiota donor on the IBD susceptibility of the recipient does not play a key role for the observed protective effect.

Another interesting question that arose during this study is whether the protective effect observed is related to the UDCA treatment that people with PSC commonly receive. Thus, we compared colitis susceptibility of germ-free wild-type mice on transfer of faecal microbiota from PBC patients, who also commonly receive UDCA, to faecal microbiota from HCs. We could not find a difference between these two groups. In addition, we transferred faecal microbiota from *Mdr2*-deficient mice, which had not received UDCA, into germ-free mice. In this set of experiments, we also observed a protective effect of the microbiota from *Mdr2*-deficient mice compared with control mice on colitis susceptibility. Therefore, our data argue against a beneficial effect of the UDCA treatment on the IBD susceptibility after FMT of PSC-IBD microbiota. However, we were not able to compare cholestatic cohorts with or without UDCA treatment and therefore, we cannot exclude an additional effect of UDCA on colitis severity mediated by the microbiota composition as it has been shown recently by He *et al*.^41^

Interestingly, we found an increased *FOXP3* mRNA and protein expression in the colon of people with active PSC-IBD, compared with active IBD without PSC. In addition, we identified an increased infiltration of Foxp3^+^ Treg cells in the inflamed colon of mice with concomitant sclerosing cholangitis in our mouse models. Interestingly, patients with genetic mutations^42^ in the *FOXP3* gene, that have no or non-functional Treg cells, develop severe intestinal inflammation. Furthermore, adoptive transfer of autologous OVA-specific or polyclonal Treg cells has been shown to reduce CD and PSC-associated UC.^43^ Therefore, our data argue for the involvement of Foxp3^+^ Treg cells in the protective effect of PSC on IBD in humans and mice. This hypothesis is supported by our finding that the protective effect of liver cholestasis on colitis severity was not detectable in the CD4^+^CD45RB^high^ T-cell transfer colitis model,^44 45^ in which Foxp3^+^ Treg cells are largely absent. One limitation of this experiment is that, although Foxp3^+^ Treg cells were depleted before the transfer into the recipient mice, there is the possibility of inducing peripheral Foxp3^+^ iTreg cells. However, colon infiltrating Foxp3^+^ Treg cells were hardly detectable in our study. Furthermore, the factors that control colonic Treg-cell accumulation during sclerosing cholangitis with concomitant colitis revealed that sclerosing cholangitis per se did not promote Treg-cell infiltration in the absence of intestinal inflammation. In fact, an increase in colonic Treg-cell accumulation was only observed in a colitogenic environment during sclerosing cholangitis. This finding is in line with a recent study by Shaw *et al* which showed that FOXP3^+^ Treg-cell frequencies gradually increase with colitis severity in intestinal biopsies of people with PSC-IBD.^46^ Nevertheless, potential differences in the suppressive capabilities of colonic FOXP3^+^ Treg cells from people with IBD and PSC-IBD have not been assessed in this study and by Shaw *et al*.^46^ Thus further studies will be essential to decipher the mechanism how PSC influences Foxp3^+^ Treg-cell expression and function in the setting of intestinal inflammation.

Overall, it remains to be elucidated what mechanism drives the increased accumulation of colonic Foxp3^+^ Treg cells during PSC-associated IBD. Beyond a participation of SCFAs in Foxp3^+^ Treg-cell differentiation in the colon, it is also tempting to speculate that the differentiation and expansion of Foxp3^+^ Treg cells already occurs in the cholestatic liver, and that consequently increased numbers of Foxp3^+^ Treg cells traffic from the liver to the colon. Indeed, increased Foxp3^+^ Treg-cell frequencies have been found in livers with different diseases like chronic viral hepatitis and hepatocellular carcinoma compared with healthy livers.^47^ Further studies will be essential to test these hypotheses.

Interestingly, it is well known that there are shared genetic risk loci between PSC and IBD, however it is also well established that the co-occurrence is far too extensive to be explained by genetics alone.^48^ Overall, our study provides novel insights into the relationship between PSC and IBD. We found that despite the common co-occurrence of both diseases, PSC can actually modify the severity of IBD to a better outcome. This effect is mediated by changes in the microbiota, which promotes the expansion of the Foxp3^+^ Treg-cell pool. A recently published report showed that IBD also ameliorates PSC.^49^ Therefore, our data suggest that disease in one organ, for example, the liver, may modify the disease in the other, for example, the intestine, in this case limiting the disease severity in both organs. Thus, we believe that our study might serve as a basis for further investigations on the molecular mechanisms underlying these processes, and could therefore lead to the discovery of novel therapeutic targets for PSC and IBD.

10.1136/gutjnl-2023-330856.supp1 Supplementary data

## Data Availability

Data are available upon reasonable request. All data relevant to the study are included in the article or uploaded as supplementary information.

## References

[1] de Vries AB , Janse M , Blokzijl H , et al . Distinctive inflammatory bowel disease phenotype in primary sclerosing cholangitis. World J Gastroenterol 2015;21:1956–71. 10.3748/wjg.v21.i6.1956 25684965 PMC4323476

[2] Rasmussen HH , Fallingborg JF , Mortensen PB , et al . Hepatobiliary dysfunction and primary sclerosing cholangitis in patients with Crohn’s disease. Scand J Gastroenterol 1997;32:604–10. 10.3109/00365529709025107 9200295

[3] Loftus EV , Harewood GC , Loftus CG , et al . PSC-IBD: a unique form of inflammatory bowel disease associated with primary sclerosing cholangitis. Gut 2005;54:91–6. 10.1136/gut.2004.046615 15591511 PMC1774346

[4] Boonstra K , Beuers U , Ponsioen CY . Epidemiology of primary sclerosing cholangitis and primary biliary cirrhosis: a systematic review. J Hepatol 2012;56:1181–8. 10.1016/j.jhep.2011.10.025 22245904

[5] Weismüller TJ , Trivedi PJ , Bergquist A , et al . Patient age, sex, and inflammatory bowel disease phenotype associate with course of primary sclerosing cholangitis. Gastroenterology 2017;152:1975–84. 10.1053/j.gastro.2017.02.038 28274849 PMC5546611

[6] Wittek A , Steglich B , Casar C , et al . A gradient of intestinal inflammation in primary Sclerosing cholangitis. Inflamm Bowel Dis 2023:izad137. 10.1093/ibd/izad137 37540889

[7] Mohammadnia-Afrouzi M , Zavaran Hosseini A , Khalili A , et al . Decrease of CD4(+) CD25(+) CD127(Low) FoxP3(+) regulatory T cells with impaired suppressive function in untreated ulcerative colitis patients. Autoimmunity 2015;48:556–61. 10.3109/08916934.2015.1070835 26333292

[8] Schwinge D , von Haxthausen F , Quaas A , et al . Dysfunction of hepatic regulatory T cells in experimental sclerosing cholangitis is related to IL-12 signaling. J Hepatol 2017;66:798–805. 10.1016/j.jhep.2016.12.001 27965154

[9] Nothnagel M , Ellinghaus D , Schreiber S , et al . A comprehensive evaluation of SNP genotype imputation. Hum Genet 2009;125:163–71. 10.1007/s00439-008-0606-5 19089453

[10] Ellinghaus D , Jostins L , Spain SL , et al . Analysis of five chronic inflammatory diseases identifies 27 new associations and highlights disease-specific patterns at shared Loci. Nat Genet 2016;48:510–8. 10.1038/ng.3528 26974007 PMC4848113

[11] Sebode M , Peiseler M , Franke B , et al . Reduced FoxP3(+) regulatory T cells in patients with primary sclerosing cholangitis are associated with Il2Ra gene Polymorphisms. J Hepatol 2014;60:1010–6. 10.1016/j.jhep.2013.12.027 24412607

[12] Goldberg R , Clough JN , Roberts LB , et al . A Crohn’s disease-associated Il2Ra enhancer variant determines the balance of T cell immunity by regulating responsiveness to IL-2 signalling. J Crohns Colitis 2021;15:2054–65. 10.1093/ecco-jcc/jjab103 34120187 PMC8684452

[13] Arpaia N , Campbell C , Fan X , et al . Metabolites produced by commensal bacteria promote peripheral regulatory T-cell generation. Nature 2013;504:451–5. 10.1038/nature12726 24226773 PMC3869884

[14] Sakaguchi S , Sakaguchi N , Asano M , et al . Immunologic self-tolerance maintained by activated T cells expressing IL-2 receptor alpha-chains (Cd25). breakdown of a single mechanism of self-tolerance causes various autoimmune diseases. J Immunol 1995;155:1151–64. 10.4049/jimmunol.155.3.1151 7636184

[15] Da Cunha T , Vaziri H , Wu GY . Primary Sclerosing cholangitis and inflammatory bowel disease: a review. J Clin Transl Hepatol 2022;10:531–42. 10.14218/JCTH.2021.00344 35836773 PMC9240248

[16] Rossen NG , Fuentes S , Boonstra K , et al . The mucosa-associated microbiota of PSC patients is characterized by low diversity and low abundance of uncultured clostridiales II. J Crohns Colitis 2015;9:342–8. 10.1093/ecco-jcc/jju023 25547975

[17] Torres J , Bao X , Goel A , et al . The features of mucosa-associated microbiota in primary sclerosing cholangitis. Aliment Pharmacol Ther 2016;43:790–801. 10.1111/apt.13552 26857969 PMC5177987

[18] Sabino J , Vieira-Silva S , Machiels K , et al . Primary Sclerosing cholangitis is characterised by intestinal dysbiosis independent from IBD. Gut 2016;65:1681–9. 10.1136/gutjnl-2015-311004 27207975 PMC5036217

[19] Kummen M , Holm K , Anmarkrud JA , et al . The gut microbial profile in patients with primary sclerosing cholangitis is distinct from patients with ulcerative colitis without biliary disease and healthy controls. Gut 2017;66:611–9. 10.1136/gutjnl-2015-310500 26887816

[20] Rühlemann MC , Heinsen F-A , Zenouzi R , et al . Faecal microbiota profiles as diagnostic biomarkers in primary sclerosing cholangitis. Gut 2017;66:753–4. 10.1136/gutjnl-2016-312180 27216937

[21] Kevans D , Tyler AD , Holm K , et al . Characterization of intestinal microbiota in ulcerative colitis patients with and without primary sclerosing cholangitis. J Crohns Colitis 2016;10:330–7. 10.1093/ecco-jcc/jjv204 26526357 PMC4957469

[22] Awoniyi M , Wang J , Ngo B , et al . Protective and aggressive bacterial subsets and metabolites modify hepatobiliary inflammation and fibrosis in a murine model of PSC. Gut 2023;72:671–85. 10.1136/gutjnl-2021-326500 35705368 PMC9751228

[23] Quraishi MN , Sergeant M , Kay G , et al . The gut-adherent microbiota of PSC-IBD is distinct to that of IBD. Gut 2017;66:386–8. 10.1136/gutjnl-2016-311915 27196590

[24] Kühn R , Löhler J , Rennick D , et al . Interleukin-10-deficient mice develop chronic enterocolitis. Cell 1993;75:263–74. 10.1016/0092-8674(93)80068-p 8402911

[25] Fickert P , Pollheimer MJ , Beuers U , et al . Characterization of animal models for primary sclerosing cholangitis (PSC). J Hepatol 2014;60:1290–303. 10.1016/j.jhep.2014.02.006 24560657 PMC4517670

[26] Roy U , Gálvez EJC , Iljazovic A , et al . Distinct microbial communities trigger colitis development upon intestinal barrier damage via innate or adaptive immune cells. Cell Rep 2017;21:994–1008. 10.1016/j.celrep.2017.09.097 29069606 PMC5668567

[27] Pose E , Sancho-Bru P , Coll M . 3,5-Diethoxycarbonyl-1,4-dihydrocollidine diet: a rodent model in cholestasis research. Methods Mol Biol 2019;1981:249–57. 10.1007/978-1-4939-9420-5_16 31016659

[28] Soukou S , Brockmann L , Bedke T , et al . Role of IL-10 receptor signaling in the function of CD4+ T-regulatory type 1 cells: T-cell therapy in patients with inflammatory bowel disease. Crit Rev Immunol 2018;38:415–31. 10.1615/CritRevImmunol.2018026850 30806217

[29] Kamanaka M , Huber S , Zenewicz LA , et al . Memory/Effector (CD45RB(Lo)) Cd4 T cells are controlled directly by IL-10 and cause IL-22-dependent intestinal pathology. J Exp Med 2011;208:1027–40. 10.1084/jem.20102149 21518800 PMC3092344

[30] Schirmer M , Garner A , Vlamakis H , et al . Microbial genes and pathways in inflammatory bowel disease. Nat Rev Microbiol 2019;17:497–511. 10.1038/s41579-019-0213-6 31249397 PMC6759048

[31] Song X , Sun X , Oh SF , et al . Microbial bile acid metabolites modulate gut Rorgamma(+) regulatory T cell homeostasis. Nature 2020;577:410–5. 10.1038/s41586-019-1865-0 31875848 PMC7274525

[32] Asseman C , Mauze S , Leach MW , et al . An essential role for interleukin 10 in the function of regulatory T cells that inhibit intestinal inflammation. J Exp Med 1999;190:995–1004. 10.1084/jem.190.7.995 10510089 PMC2195650

[33] Rühlemann M , Liwinski T , Heinsen F-A , et al . Consistent alterations in faecal microbiomes of patients with primary sclerosing cholangitis independent of associated colitis. Aliment Pharmacol Ther 2019;50:580–9. 10.1111/apt.15375 31250469 PMC6899739

[34] Vacca M , Celano G , Calabrese FM , et al . The controversial role of human gut lachnospiraceae. Microorganisms 2020;8:573. 10.3390/microorganisms8040573 32326636 PMC7232163

[35] Parada Venegas D , De la Fuente MK , Landskron G , et al . Short chain fatty acids (Scfas)-mediated gut epithelial and immune regulation and its relevance for inflammatory bowel diseases. Front Immunol 2019;10:277. 10.3389/fimmu.2019.00277 30915065 PMC6421268

[36] Smith PM , Howitt MR , Panikov N , et al . The microbial metabolites, short-chain fatty acids, regulate colonic Treg cell homeostasis. Science 2013;341:569–73. 10.1126/science.1241165 23828891 PMC3807819

[37] Kim CH . Control of lymphocyte functions by gut microbiota-derived short-chain fatty acids. Cell Mol Immunol 2021;18:1161–71. 10.1038/s41423-020-00625-0 33850311 PMC8093302

[38] Furusawa Y , Obata Y , Fukuda S , et al . Commensal microbe-derived butyrate induces the differentiation of colonic regulatory T cells. Nature 2013;504:446–50. 10.1038/nature12721 24226770

[39] Liao L , Schneider KM , Galvez EJC , et al . Intestinal dysbiosis augments liver disease progression via NLRP3 in a murine model of primary Sclerosing cholangitis. Gut 2019;68:1477–92. 10.1136/gutjnl-2018-316670 30872395

[40] Nakamoto N , Sasaki N , Aoki R , et al . Gut Pathobionts underlie intestinal barrier dysfunction and liver T helper 17 cell immune response in primary sclerosing cholangitis. Nat Microbiol 2019;4:492–503. 10.1038/s41564-018-0333-1 30643240

[41] He Q , Wu J , Ke J , et al . Therapeutic role of ursodeoxycholic acid in colitis-associated cancer via gut microbiota modulation. Mol Ther 2023;31:585–98. 10.1016/j.ymthe.2022.10.014 38556635 PMC9931610

[42] Bacchetta R , Passerini L , Gambineri E , et al . Defective regulatory and effector T cell functions in patients with FoxP3 mutations. J Clin Invest 2006;116:1713–22. 10.1172/JCI25112 16741580 PMC1472239

[43] Desreumaux P , Foussat A , Allez M , et al . Safety and efficacy of antigen-specific regulatory T-cell therapy for patients with refractory Crohn’s disease. Gastroenterology 2012;143:1207–17. 10.1053/j.gastro.2012.07.116 22885333

[44] Powrie F , Leach MW , Mauze S , et al . Phenotypically distinct subsets of CD4+ T cells induce or protect from chronic intestinal inflammation in C. B-17 Scid mice. Int Immunol 1993;5:1461–71. 10.1093/intimm/5.11.1461 7903159

[45] Powrie F , Correa-Oliveira R , Mauze S , et al . Regulatory interactions between CD45RBhigh and CD45RBlow CD4+ T cells are important for the balance between protective and pathogenic cell-mediated immunity. J Exp Med 1994;179:589–600. 10.1084/jem.179.2.589 7905019 PMC2191378

[46] Shaw DG , Aguirre-Gamboa R , Vieira MC , et al . Antigen-driven colonic inflammation is associated with development of dysplasia in primary sclerosing cholangitis. Nat Med 2023;29:1520–9. 10.1038/s41591-023-02372-x 37322120 PMC10287559

[47] Oo YH , Weston CJ , Lalor PF , et al . Distinct roles for CCR4 and CXCR3 in the recruitment and positioning of regulatory T cells in the inflamed human liver. J Immunol 2010;184:2886–98. 10.4049/jimmunol.0901216 20164417

[48] Ji S-G , Juran BD , Mucha S , et al . Genome-wide association study of primary sclerosing cholangitis identifies new risk Loci and Quantifies the genetic relationship with inflammatory bowel disease. Nat Genet 2017;49:269–73. 10.1038/ng.3745 27992413 PMC5540332

[49] Gui W , Hole MJ , Molinaro A , et al . Colitis ameliorates cholestatic liver disease via suppression of bile acid synthesis. Nat Commun 2023;14:3304. 10.1038/s41467-023-38840-8 37280200 PMC10244448

