# Supplementary material for: Protective function of sclerosing cholangitis on IBD
Source: Gut. 2024 Jun 5;73(8):1292–301. doi: 10.1136/gutjnl-2023-330856 (PMC11287650; doi:10.1136/gutjnl-2023-330856)
Supplement: Supplementary data [file gutjnl-2023-330856supp002.pdf]

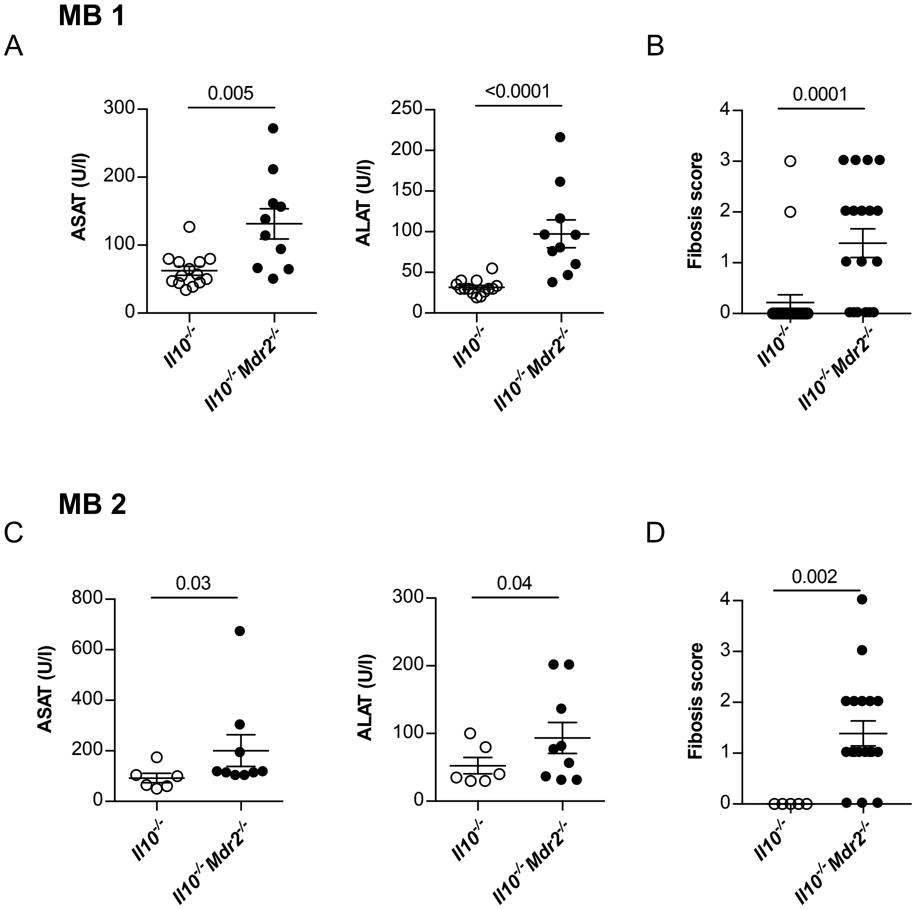

Figure S1: Liver inflammation in *Il10-/-* mice with concomitant *Mdr2-/-* induced cholangitis. Mice were bred under SPF conditions of the local mouse facility (MB1), as outlined in Figure 1. At 12 weeks of age, mice were sacrificed and liver pathology was assessed by (A) serum Aspartate Aminotransferase (ASAT) and Alanine Aminotransferase (ALAT) (n=14 *Il10-/-*, n=10 *Il10-/-Mdr2-/-*), as described in the material and methods. (B) Fibrosis score was analyzed by Sirius Red staining. (C) Mice bred in the presence of colitogenic MB2, were sacrificed and liver pathology was analyzed based on serum Aspartate Aminotransferase (ASAT) and Alanine Aminotransferase (ALAT) (n=6 *Il10-/-*, n=9 *Il10-/-Mdr2-/-*). (D) Fibrosis score was analyzed by Sirius Red staining. In all experiments, Mann-Whitney U test was performed for statistical analysis.

Figure S1

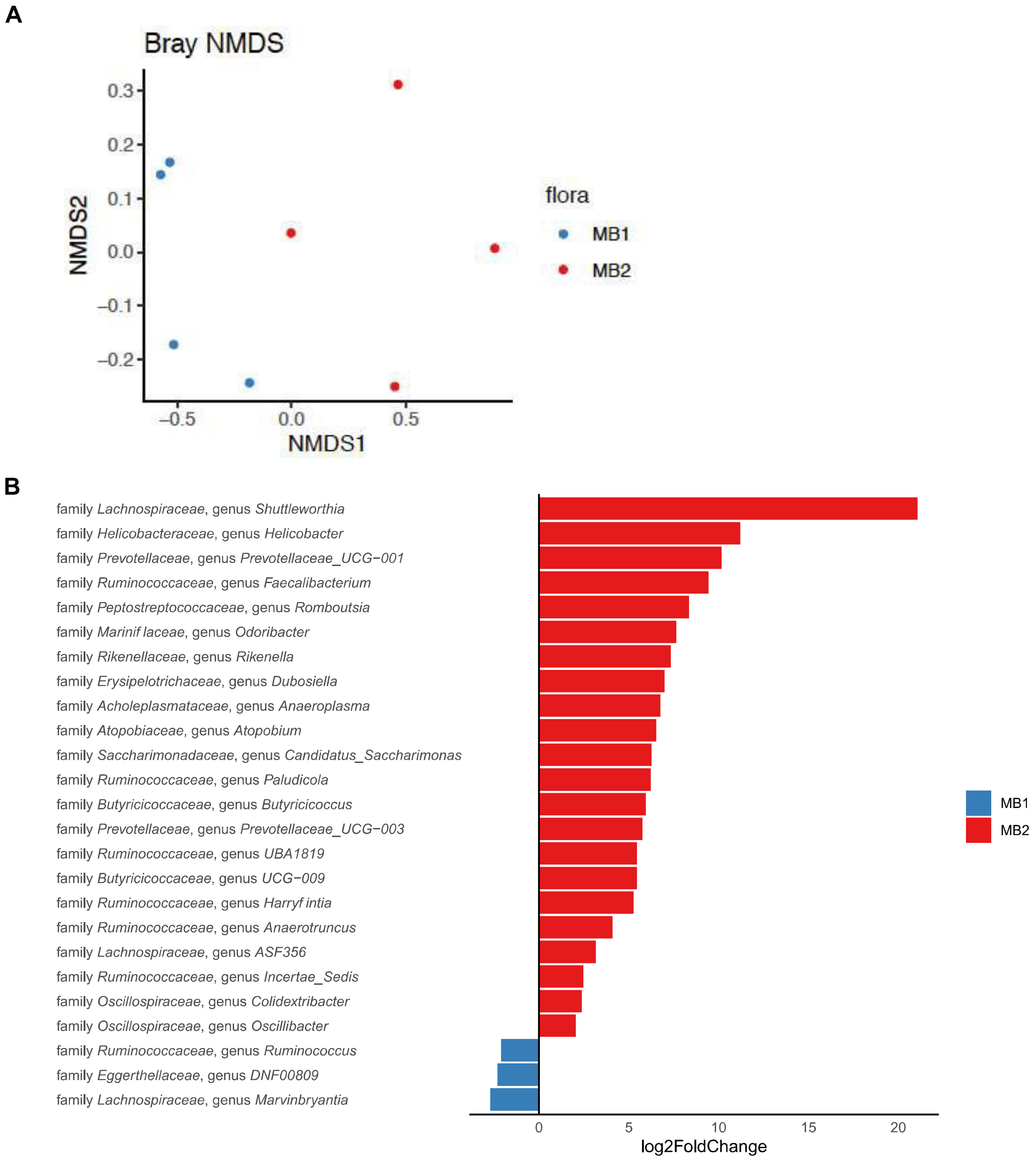

Figure S2: Microbiota profiling of MB1 and in colitogenic MB2. Wild-type mice bred under microbiota conditions 1 (MB1) and 2 (MB2) were sacrificed and stool samples from 4 mice were collected per group. Microbiota profiling was performed as described in the materials and methods. A) PCoA of Bray-Curtis dissimilarities shows beta diversity across mice bred under MB1 and MB2 conditions. (B) Genera with significantly different abundance between groups.

Figure S2

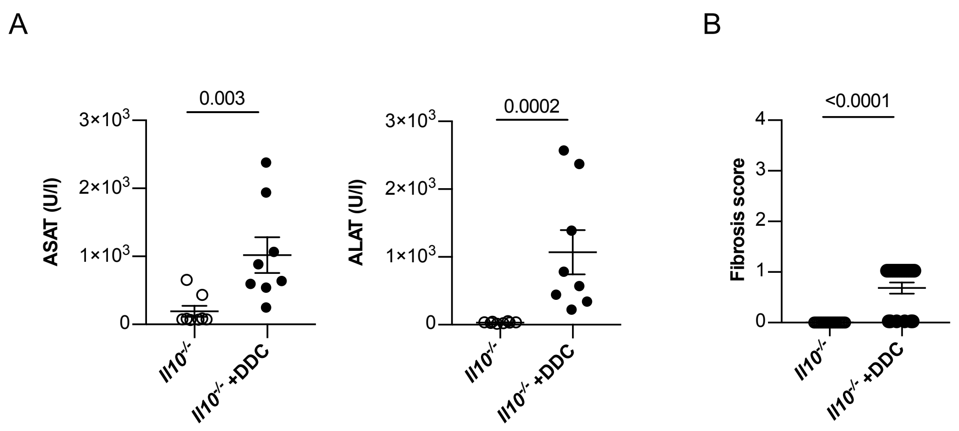

Figure S3: Liver inflammation in *Il10-/-* mice after DDC-induced liver cholestasis. Liver cholestasis was induced by 2% DDC feeding in *Il10-/-* mice gavaged with MB2, as described in Figure 2. After 9 days of feeding the mice with the DDC diet, mice were sacrificed and liver inflammation was analyzed by (A) serum Aspartate Aminotransferase (ASAT) and Alanine Aminotransferase (ALAT) (8= mice per group). (B) Fibrosis score was analyzed by Sirius Red staining. For statistical analysis, Mann-Whitney U test was performed.

Figure S3

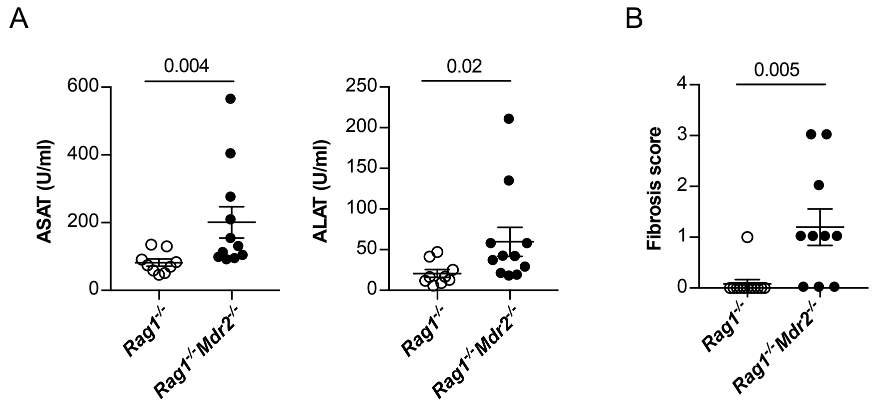

Figure S4: Liver inflammation in *Rag1-/-Mdr2-/-* mice after induction of Foxp3-CD45RBhigh transfer colitis. Colitis was induced in *Rag1-/-* and *Rag1-/-Mdr2-/-* mice upon transferring Foxp3-CD45RBhigh cells, as described in Figure 3. On day 14, mice were sacrificed and liver inflammation was analyzed by (A) serum Aspartate Aminotransferase (ASAT) and Alanine Aminotransferase (ALAT) (n=9 *Rag1-/-* n=11 *Rag1-/-Mdr2-/-*). (B) Fibrosis score was analyzed by Sirius Red staining. For statistical analysis, Mann-Whitney U test was performed.

Figure S4

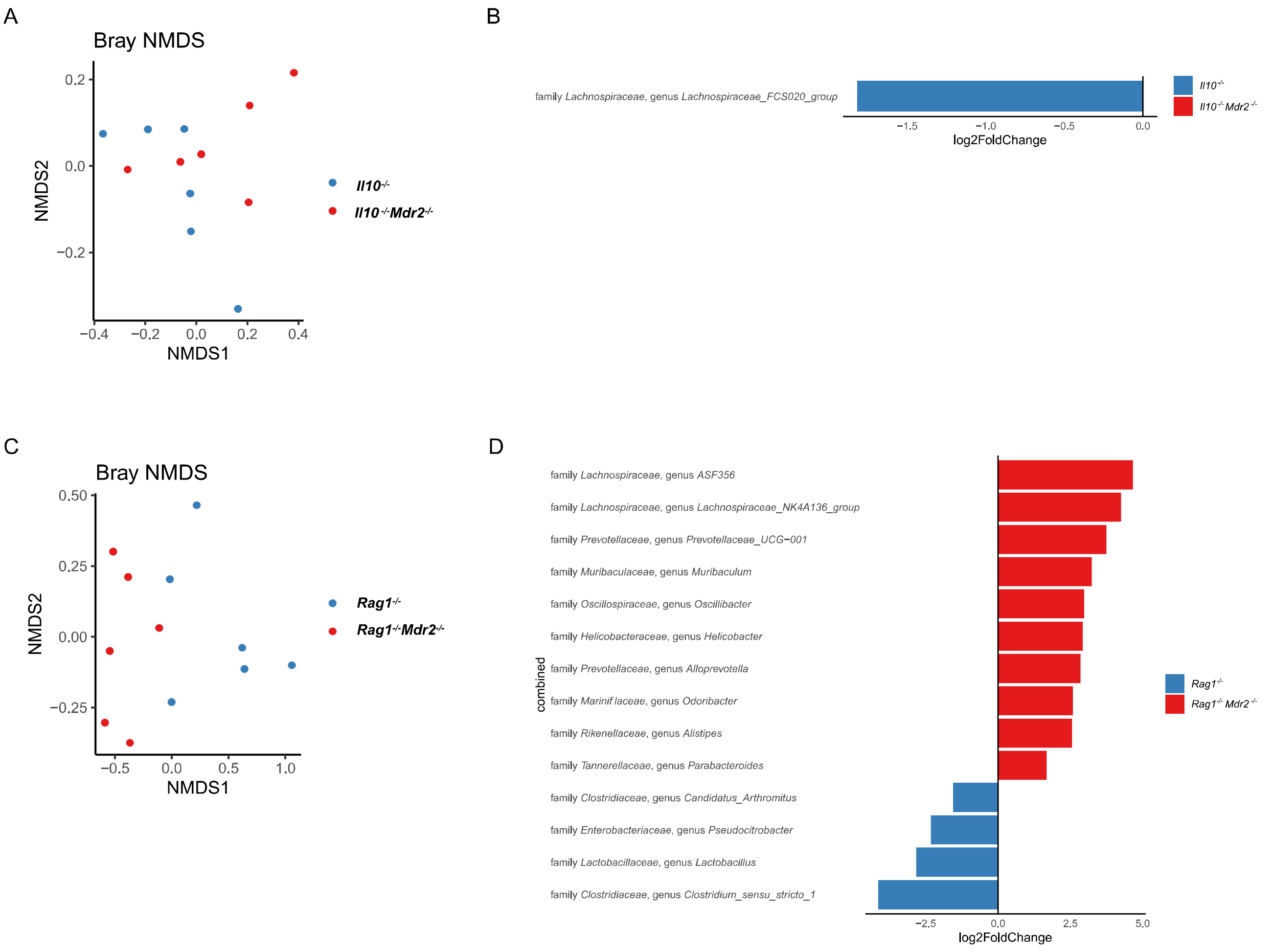

Figure S5: Concomitant liver inflammation alters the intestinal microbiota in *Il10-/-* and *Rag1-/-* mice suffering from colitis. *Il10-/-*, *Il10-/-Mdr2-/-* mice (MB1) and *Rag1-/-*, *Rag1-/-Mdr2-/-* mice (MB2) were sacrificed as described in Figures 1 and 3, respectively, and stool samples from 6 mice were collected per group. Microbiota profiling was performed as described in the materials and methods. (A) PCoA of Bray-Curtis dissimilarities shows beta diversity across *Il10-/-* and *Il10-/-Mdr2-/-* mice. (B) Genera with significantly different abundances between groups. (C) PCoA of Bray-Curtis dissimilarities shows beta diversity across *Rag1-/-* and *Rag1-/-Mdr2-/-* mice. (D) Genera with significantly different abundances between both groups.

Figure S5

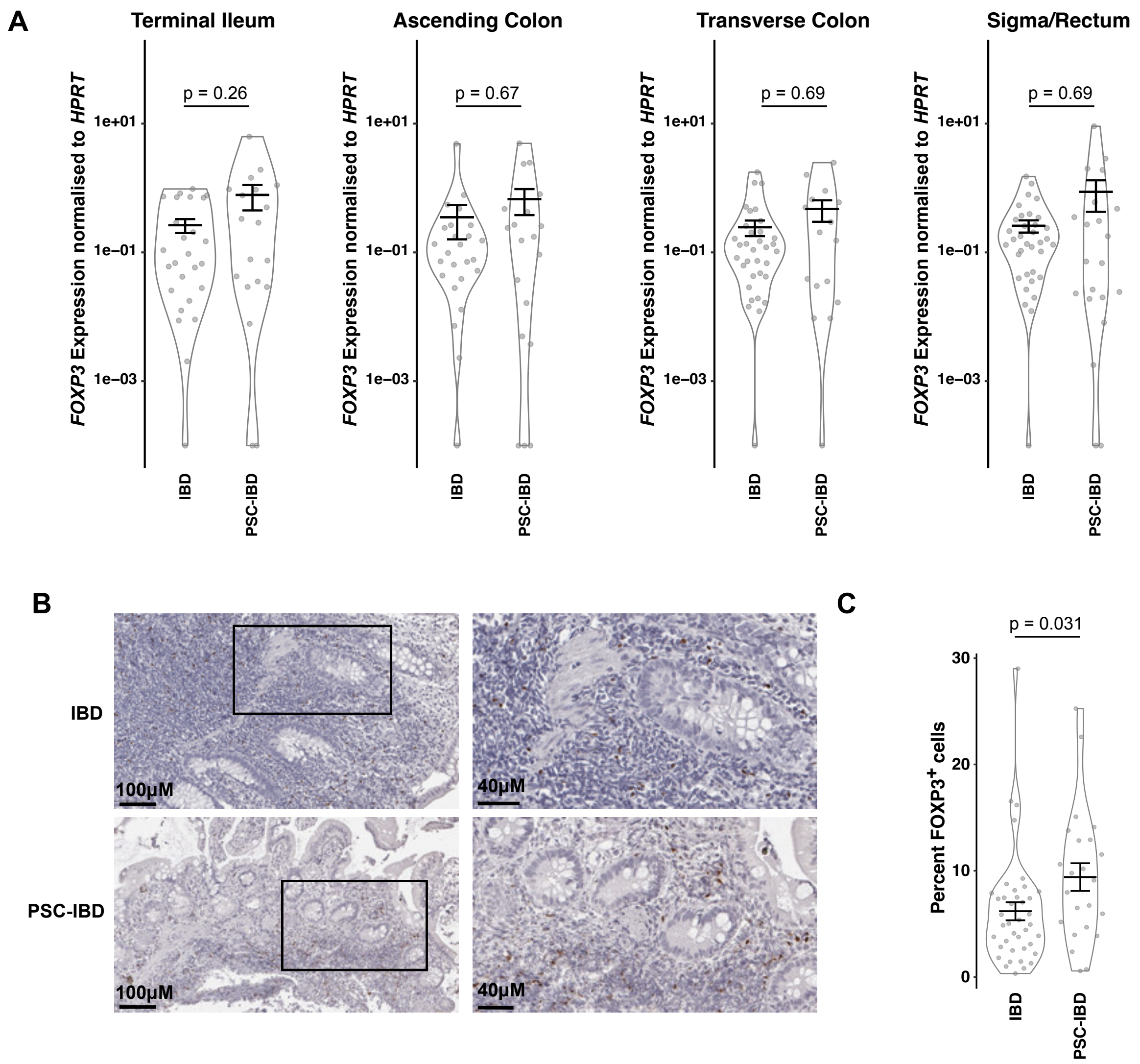

Figure S6: Increased FOXP3 protein expression in intestinal biopsies of people with PSC-IBD. (A) *FOXP3* mRNA expression levels were analyzed from intestinal biopsies taken from the terminal ileum, ascending colon, descending colon or sigma/rectum from every person with clinically active disease. (B) Representative images and (C) quantification of FOXP3+ cells in intestinal biopsies taken from the terminal ileum and sigma/rectum from every person with clinically active disease. To test for significance MLEM, post hoc Dunnett test was used.

Figure S6

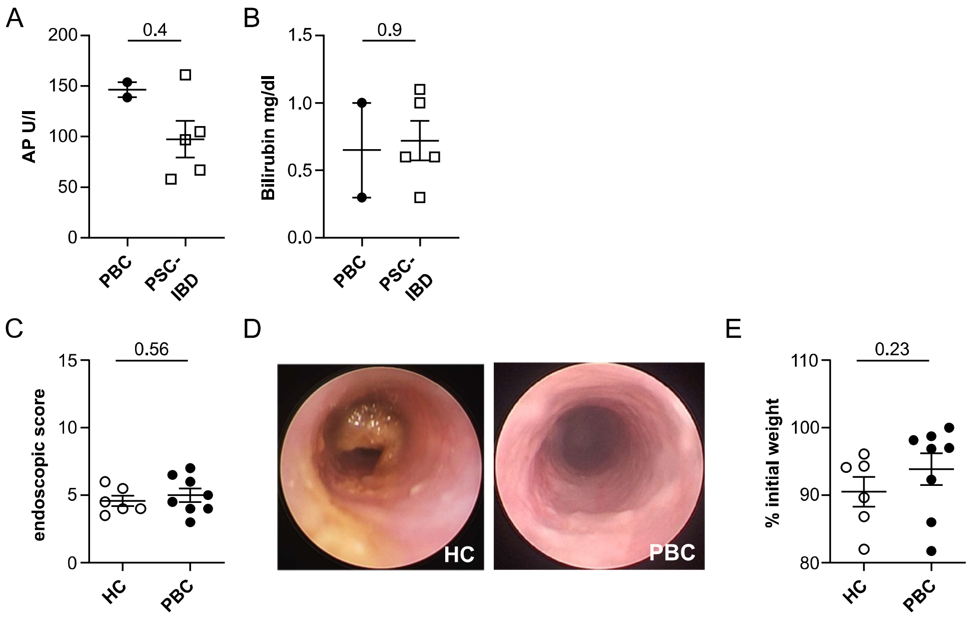

Figure S7: Colitis severity in germ-free mice is not affected by UDCA treatment of the stool donor. (A+B) People with PBC with comparable cholestasis to people with PSC-IBD were chosen. Stool transfer from healthy control (HC) and people with primary biliary cholangitis (PBC) that received UDCA treatment into germ-free mice before the chemical induction of colitis for 7 days. On day 9, colonic inflammation was analyzed by (C+D) colonoscopy as represented by endoscopic score and representative pictures and (E) weight loss. Each dot represents one mouse. For statistical analysis Mann-Whitney U test was performed.

Figure S7

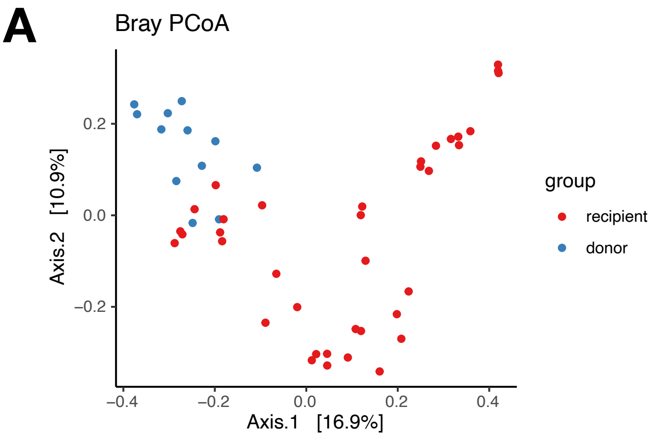

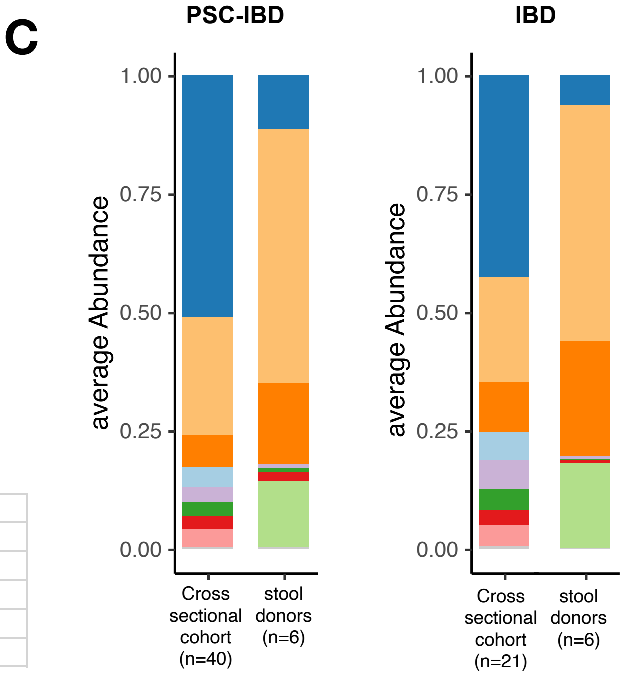

B

	Df	SumOfSqs	R2	F	Pr(>F)
disease	1	0.6848	0.04027	3.5182	0.001
group	1	1.671	0.09826	8.5848	0.001
donor	10	7.6424	0.44941	3.9263	0.001
Residual	36	7.0073	0.41206		
Total	48	17.0055	1		

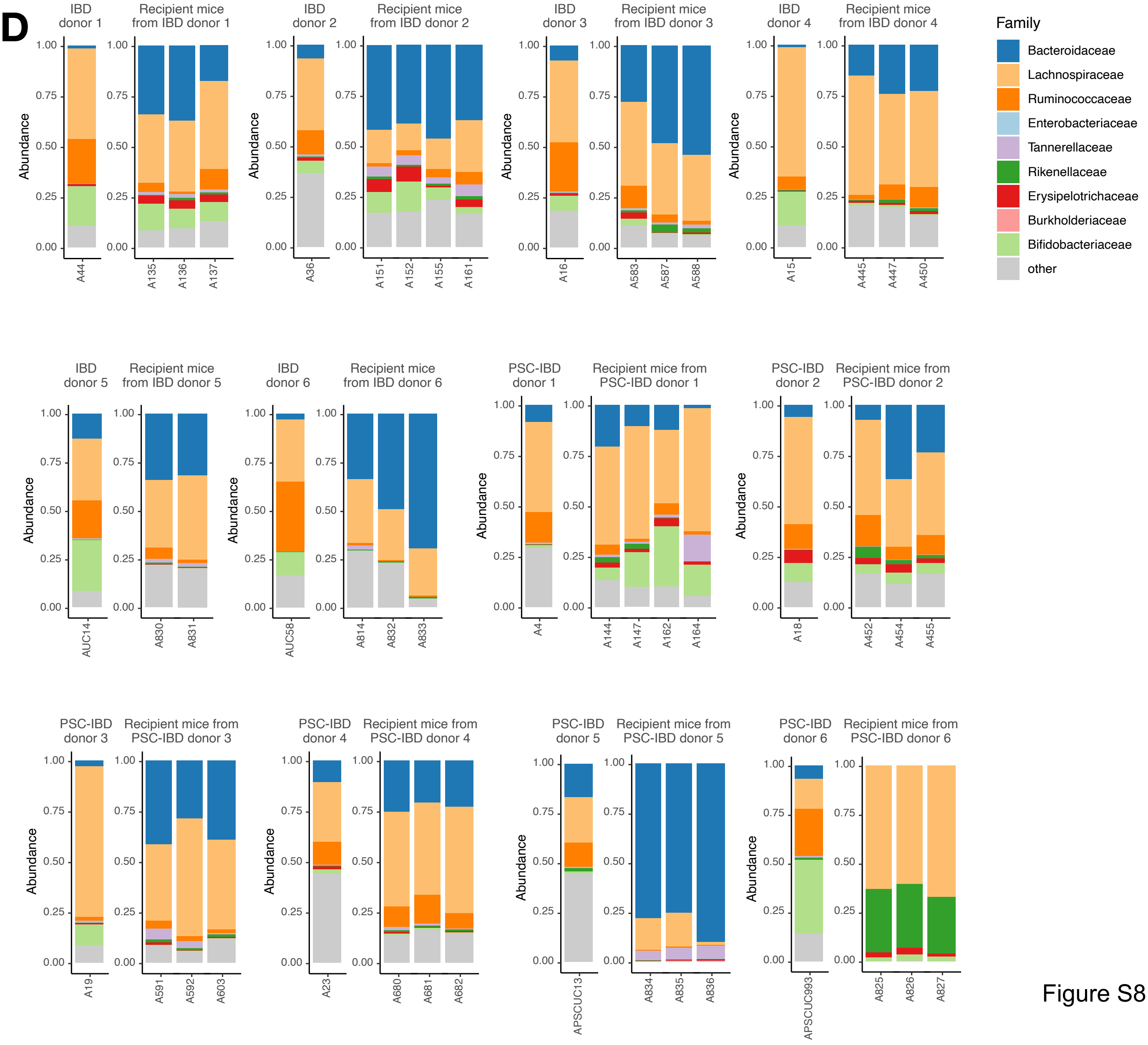

Figure S8

Figure S8: Microbiota profiling of stool from human donors and respective recipient mice. (A) PCoA of Bray-Curtis dissimilarities shows beta diversity across stool samples from human donors and recipient mice. (B) PERMANOVA analysis showing the contribution of disease, group (donor vs. recipient) and donor to the variation observed in the data shown in A. (C) A bar plot displaying the most abundant families, comparing a cross-section with the stool donors used in this study. (D) Abundance of the 10 highest abundant families across donor samples and their respective recipient mouse samples.
