# Supplementary material for: Protective function of sclerosing cholangitis on IBD
Source: Gut. 2024 Jun 5;73(8):1292–301. doi: 10.1136/gutjnl-2023-330856 (PMC11287650; doi:10.1136/gutjnl-2023-330856)
Supplement: Supplementary data [file gutjnl-2023-330856supp004.pdf]

Table S2. Characteristics of patients analyzed by immunohistochemistry

	IBD	PSC-IBD
n-value	30	19
age in years	42 ± 14.5	37 ± 14.5
sex male %	60%	84%
**Disease activity, n (%)**		
Remission	12 (40%)	9 (47%)
Mild	13 (43%)	7 (37%)
Moderate	3 (10%)	3 (16%)
Severe	2 (7%)	0
**Medications, n (%)**		
5-ASA	17 (57%)	6 (32%)
Thiopurines	2 (7%)	7 (37%)
Anti-TNF	14 (47%)	5 (26%)
Anti-IL12/23	1 (3%)	2 (11%)
UDCA	0	16 (84%)
Methotrexate	1 (3%)	0
