# Supplementary material for: Protective function of sclerosing cholangitis on IBD
Source: Gut. 2024 Jun 5;73(8):1292–301. doi: 10.1136/gutjnl-2023-330856 (PMC11287650; doi:10.1136/gutjnl-2023-330856)
Supplement: Supplementary data [file gutjnl-2023-330856supp003.pdf]

Table S1. Characteristics of microbiota donors.

	IBD	PSC-IBD	PBC
n-value	6	5	2
age in years	41±15.2	43±9.7	65±2.2
sex male (%)	4 (66%)	3 (60%)	2 (100%)
**IBD disease activity**, **n (%)**			
Remission	2 (33%)	3 (60%)	0 (0%)
Mild	2 (33%)	0 (0%)	0 (0%)
Moderate	1 (16%)	2 (40%)	0 (0%)
Severe	1 (16%)	0 (0%)	0 (0%)
**Medications, n (%)**			
5-ASA	4 (66%)	3 (60%)	0 (0%)
Thiopurines	2 (33%)	1 (20%)	0 (0%)
Anti-TNF	1 (16%)	2 (40%)	0 (0%)
Anti-IL12/23	1 (16%)	0 (0%)	0 (0%)
UDCA	0	5/5 (100%)	2/2 (100%)
Methotrexate	1 (16%)	0 (0%)	0 (0%)

*IBD disease activity was categorized based on Mayo score for UC (remission: 0-2, mild: 3-5, moderate: 6-10, severe 11-12 points).
