# Supplementary material for: Protective function of sclerosing cholangitis on IBD
Source: Gut. 2024 Jun 5;73(8):1292–301. doi: 10.1136/gutjnl-2023-330856 (PMC11287650; doi:10.1136/gutjnl-2023-330856)
Supplement: Supplementary data [file gutjnl-2023-330856supp001.pdf]

Extended methods

Mice

Rag1$^{-/-}$ mice, *Foxp3*^{RFP}*Ifng*^{Katushka}*Il17a*^{GFP} reporter mice, *Il10*$^{-/-}$mice, *Mdr2*$^{-/-}$mice, *Il10*$^{-/-}$*Mdr2*$^{-/-}$mice, and *Rag1*$^{-/-}$*Mdr2*$^{-/-}$ mice were bred and housed under specific pathogen-free conditions (SPF) at the animal facility of the University Hospital Hamburg-Eppendorf. *Il10*$^{-/-}$*Mdr2*$^{-/+}$ were crossed with each other in order to obtain *Il10*$^{-/-}$ and *Il10*$^{-/-}$*Mdr2*$^{-/-}$ littermates. These mice were then separated with respect to their genotype after weaning. This way the mice could develop a specific microbiota dependent on the genotype. *Il10*$^{-/-}$*Mdr2*$^{-/+}$ mice were bred with two different microbiomes, one that does not induce spontaneous colitis (MB1) and a colitogenic microbiome (MB2), containing *Helicobacter hepaticus*(24). Littermates were separated with respect to their genotype. C57BL/6 wild-type mice were bred and housed under germ-free conditions at the UKE, Hamburg. All animals were housed under a 12h dark/light cycle with an ambient temperature of 22°C ± 1°C and 50% ± 5% relative humidity. Food and water were provided *ad libitum*. Male and female 13 ±1 week old littermates were used for all experiments. Animal experiments were approved by the local ethics committee (N17/2012, N39/2021, N54/2022, N95/2023).

Human studies

Intestinal biopsies were taken from people undergoing colonoscopy at University Hospital Hamburg-Eppendorf. We collected 2 paired biopsies at 4 sampling sites in the colon and terminal ileum. One was used for RNA extraction while the other was used for microbiota profiling. The biopsies were snap-frozen in liquid nitrogen directly after colonoscopy and stored at −80°C until processing. For detailed patient information (age, BMI, years of diagnosis, smoking, gender, IBD activity and medication) see Wittek et al, 2023. Patients were included, if older than 18 years and without antibiotics treatment for 6 months prior to endoscopy. Patients with infectious colitis, celiac disease, or confirmed pregnancy were excluded. Disease severity was based on Mayo score for UC (remission: 0-2, mild: 3-5, moderate: 6-10, severe 11-12 points) and Harvey-Bradshaw index for CD (remission: 0-4, mild: 5-7, moderate: 8-16, severe >16 points). For comparison of IBD patients we assigned scores for disease severity (remission: 0, mild: 1, moderate: 2, severe 3 points)(25). Human studies were approved by the local ethical committee (Ethik Kommission der Ärztekammer Hamburg

Bedke T, *et al. Gut* 2024; 73:1292–1301. doi: 10.1136/gutjnl-2023-330856

PV4444, PV7106). Patients or the public were not involved in the design, or conduct, or reporting, or dissemination plans of our research.

DDC-induced sclerosing cholangitis

The chemically-induced model for experimental sclerosing cholangitis, 3,5-diethoxycarbonyl-1,4-dihydrocollidine (DDC; Merk, Germany) was used, by adding 0.1 % DDC *w/w* to the diet. *Il10*$^{-/-}$ mice at 10–14-weeks old were fed for 8 days *ad libitum* with a DDC diet. At day 8, mouse development of intestinal inflammation was assessed by endoscopy. One day later, mice were sacrificed and organs were processed as indicated.

Transfer colitis

Lymphocytes were isolated from the spleen and lymph nodes of *Foxp3*^{RFP}*Il17a*^{Katushka}*Il10*^{GFP} reporter mice, and CD4^{+} T cells were pre-enriched using MACS according to the manufacturer's instructions (Miltenyi Biotech, Bergisch-Gladbach, Germany). Naïve CD4^{+} CD45RB^{high} *Foxp3*$^{RFP-}$ T cells were fluorescence activated cell sorted after incubation with anti-mouse CD4-PacBlue and CD45RB-AF647 fluorochrome-labeled antibodies (both Biolegend, London, England) using theAria III device (BD Biosciences, Heidelberg, Germany). To induce colitis, 2x10^{4} Naïve CD4^{+} CD45RB^{high} *Foxp3*$^{RFP-}$ T cells were injected intraperitoneally into *Rag1*$^{-/-}$ and *Rag1*$^{-/-}$*Mdr2*$^{-/-}$ mice. Mice were monitored for development of intestinal inflammation by weight loss and endoscopy.

Anti-IL-10 receptor antibody-induced colitis

Mice were each injected with 250 µg of anti-IL10 receptor-alpha (anti-IL10Ra; clone: 1B1, source: HHMI, R.A. Flavell) twice a week intraperitoneally. Colitis development was monitored by weight loss and endoscopy. Thirteen days after the first injection, mice were sacrificed and analyzed for pathological conditions of the intestine.

DSS colitis

Mice received drinking water supplemented with 2% DSS for 7 days, followed by 2 days of pure drinking water in the absence of DSS, to induce acute DSS colitis (DSS m.w.: 36.000–50.000; MP Biomedicals, Illkirch, France). Colitis development was

Bedke T, *et al. Gut* 2024; 73:1292–1301. doi: 10.1136/gutjnl-2023-330856

monitored by endoscopy. The mice were sacrificed and analyzed for pathological conditions of the intestine.

Endoscopy

Colonoscopy was performed at the indicated time points to monitor the severity of intestinal inflammation as described before (Becker *et al.,* 2006) using the Coloview System (Karl Storz, Germany). In brief, anesthetized mice were endoscopically scored concerning 5 parameters: thickening of the colon, changes in vascular pattern, granularity of the mucosal surface, stool consistency, and visible fibrin, each graded 1 to 3, resulting in an overall score between 0 (healthy) and 15 (severe colitis).

Fecal microbiota transplantation

For murine fecal transplantation, donor mice with a colitogenic microbiome (MB2) were sacrificed and stool was collected from the colon, including the caecum, directly into thioglycolate medium (Merck, Darmstadt, Germany). Pooled samples from a minimum of 5 mice were smashed through a 70 µm cell strainer, frozen, and stored at -80°C. Upon use, stool samples were thawed, transferred to BHI medium (Merck, Darmstadt, Germany), and centrifuged for 10 min at 500g. The supernatant was resuspended in BHI medium and immediately gavaged. Each mouse was gavaged with 200 ul of stool. For human FMT, stool samples from people with IBD and PSC-IBD, coming to the clinic for routine care appointments were collected and frozen in 20% glycerol and processed for transfer into germ-free mice as described above.

Taxonomic microbiota analysis

Fresh stool samples of humans and mice were collected and immediately stored at −20°C until analysis. DNA was extracted according to established protocols using a method combining mechanical disruption (bead-beating) and phenol/chloroform-based purification previously described (Turnbaugh *et al., 2009*). Briefly, a sample was suspended in a solution containing 500 µL of extraction buffer (200 mM Tris, 20 mM EDTA, and 200 mM NaCl [pH 8.0]), 200 µL of 20% SDS, 500 µL of phenol:chloroform:isoamyl alcohol (24:24:1). Samples were homogenized twice with a bead beater for 2 min. After precipitation of DNA, crude DNA extracts were resuspended in Tris, EDTA (TE) buffer. Amplification of the V3-V4 region of the 16S

Bedke T, *et al. Gut* 2024; 73:1292–1301. doi: 10.1136/gutjnl-2023-330856

rRNA gene was performed according to previously described protocols. Samples were sequenced on an Illumina NovaSeq platform (PE250).
Microbiota profiling of adherent microbiota of human intestinal biopsies was carried out as described (Wittek *et al.,* 2023). Briefly, DNA was extracted using the DNeasy Blood & Tissue Kit, followed by amplification of variable regions V1 and V2 of the 16S rRNA gene. PCR products were verified and quantified before pooling and sequencing on the Illumina MiSeq v3 2x300bp. Demultiplexing after sequencing was based on 0 mismatches in the barcode sequences. We processed both data sets with the same pipeline (dada2, pyhloseq, DESeq2) and reference data base (Silva). Where sequencing occurred in two batches, we included the batch variable in the design formula during DESeq2 analysis. Importantly, in both cases we sequenced all groups to be compared with the same method.

Transaminases

To monitor liver damage, aspartate aminotransferase (ASAT) and alanine aminotransferase (ALAT) were analyzed in blood serum at the Institute for Experimental Immunology and Hepatology (UKE, Hamburg), using an automated procedure (COBAS MIRA; Roche, Basel, Switzerland).

Cell isolation

Mice were sacrificed by CO_2 and O_2 and immediately perfused with 5mL PBS via the left heart ventricle. Colons were harvested, rinsed in PBS, cut into small pieces, and incubated in a buffer containing 1.5% DTT (AppliChem, Darmstadt, Germany) for 20 min at 37°C. The resulting cell suspension, including intraepithelial cells (IEL), was collected. In a second step, lamina propria cells were isolated from the remaining tissue using collagenase solution containing 100U/ml collagenase (Sigma-Aldrich, Taufkirchen, Germany) and 1000U/ml Dnase I (AppliChem, Darmstadt, Germany). Following a 45 min incubation step at 37°C, the content was smashed through a 100 µm cell strainer and pooled with IELs. Tissue homogenates were washed with PBS + 1% FBS at 380g and 4°C for 10 min. Leukocytes were isolated using a Percoll gradient (GE Healthcare, Uppsala, Sweden). After isolation, cells were processed as indicated.

Flow Cytometry

For surface staining, the cells were incubated with the following fluorochrome-conjugated monoclonal antibodies: anti-CD45 (clone: 30F11), anti-CD3 (clone: 17A2), anti-CD4 (clone: RM4-5), and anti-CD45RB (clone:C3 63-16A) in the presence of a blocking anti-FcgR mAb (clone: 2.4G2) for 20 min at 4°C. Unless otherwise specified, mAbs were purchased from Biolegend (London, England).

For intracellular Foxp3 expression, cell surface markers were stained as described above, followed by fixation of bound antibodies with 4% formalin for 30 min and permeabilization with 0.1% NP-40 for 4 min both at RT. For detection, cells were incubated with the PE-conjugated Foxp3 mAb (clone: JES5-16E3, eBioscience) overnight at 4°C. Cells were analyzed using a Fortessa flow cytometer (BD Biosciences) and FlowJo software (Tree Star, Ashland, OR, USA).

RNA extraction and Real-Time PCR analysis

Total RNA was extracted from intestinal biopsies using Trizol Reagent (Invitrogen, Waltham MA) and bead beating as previously described (Pelczar *et al.,* 2016). For cDNA synthesis, we used the High-Capacity cDNA Reverse Transcription Kit (Thermofisher) on 2mg of RNA per reaction, following the manufacturer’s instructions. For Real-time PCR (RT-PCR) analysis, TaqMan Fast Advanced Master Mix (Thermofisher) was used. The following TaqMan Probes were used: *FOXP3* (Hs01058534_m1) and *HPRT1* (Hs02800695_m1). Relative expression was normalized to HPRT and calculated using the 2^{-DDCt} method. For significance testing, we applied a linear mixed-effects model using the lme function in the nlme R package (version 3.1) with the patient ID included as a random effect. This was followed by post-hoc testing with Dunnett’s multiple comparisons, using the glht function of the multcomp R package.

Immunohistochemistry

Immunohistochemistry was performed on 5µm formalin fixed and paraffin embedded sections of human colonic biopsies. Slides were deparaffinized and exposed to heat-induced antigen retrieval for 5 minutes in an autoclave at 121°C in pH 7.8 wash buffer (Dako, Glostrup, Denmark) and primary antibody specific for FOXP3 (dilution 1:72) was applied. Bound antibody was then visualized using the EnVision Kit (Dako). All sections were counterstained with hematoxylin. $FOXP3^+$ cells were counted in a

blinded fashion from at least 5 areas of a given histological section and divided by the total tissue surface obtained from the 5 areas.

Microbiota data analysis

16S sequencing reads from mouse stool samples were processed, aligned, and quantified to the level of amplicon sequence variants (ASVs) using the dada2 (version 1.12.1) pipeline for paired reads (Callahan *et al.,* 2016). Forward and reverse reads were trimmed to 220bp. Trimmed reads with more than 2 expected errors were discarded. After merging forward and reverse reads, chimera removal was performed. We kept all ASVs that were observed in at least 1% of all samples. Taxonomic assignment up to the genus level was performed using the SILVA database from September 2019 (v138) (Quast *et al.,* 2012).

Further analysis of ASV count tables was carried out using phyloseq (version 1.28.0) (McMurdie *et al.,* 2013). For beta diversity plots, non-metric multidimensional scaling (NMDS) was applied using Bray-Curtis dissimilarity. To find differentially abundant ASVs between groups, DESeq2 (version 1.36) (Love *et al.,* 2014) was used using Wald statistics and parametric fitting of dispersions, followed by log2 fold change shrinkage, and adjustment for multiple testing. ASVs were then filtered to an adjusted p-value < 0.05, an absolute log2 fold change > 1, and being detected in at least 3 samples.

Microbiota profiling of adherent microbiota of human intestinal biopsies was carried out as described in detail in Wittek *et al.,* 2023 following a similar pipeline as for the mouse stool samples. Importantly, reads from individual ASVs were pooled on the genus level to account for the reduced abundance of reads obtained from biopsies compared to stool.

Statistical analysis

For detailed statistical analysis of microbiota data, see microbiota data analysis above. Statistical analysis of all other data was performed with the GraphPad Prism® Software (GraphPad Software, San Diego, CA, USA). Non-parametric two-sided Mann–Whitney test was used. The significance level alpha was set to 0.05.

Bedke T, *et al. Gut* 2024; 73:1292–1301. doi: 10.1136/gutjnl-2023-330856
